# Early-stage multi-cancer detection through a plasma extracellular vesicle protein signature

**DOI:** 10.1016/j.xcrm.2026.102694

**Published:** 2026-03-24

**Authors:** Richard J. Lobb, Quan Zhou, David Fielding, Kekoolani S. Visan, Alain Wuethrich, Jing Wang, Youran Hu, Emma L. Norris, Marcus L. Hastie, Sarah Everitt, Brielle Parris, Lauren G. Aoude, Vanessa F. Bonazzi, Elizabeth Nixon, Jennifer Mooi, Kevin M. Koo, Kenneth O'Byrne, Arutha Kulasinghe, Rayleen V. Bowman, Ian A. Yang, Niall M. Corcoran, Christopher M. Hovens, Michael MacManus, Jeffrey J. Gorman, Bryan W. Day, Gunter Hartel, David C. Whiteman, Niall Tebbutt, John M. Mariadason, Kwun M. Fong, Andrew P. Barbour, Matt Trau, Andreas Möller

**Affiliations:** 1 Australian Institute for Bioengineering and Nanotechnology (AIBN), The University of Queensland, Brisbane, QLD 4072, Australia; 2 Tumour Microenvironment Laboratory, QIMR Berghofer Medical Research Institute, Brisbane, QLD 4006, Australia; 3 Department of Thoracic Medicine, Royal Brisbane and Women’s Hospital, Brisbane, QLD 4029, Australia; 4 JC STEM Lab, Department of Otorhinolaryngology, Chinese University of Hong Kong, Shatin, Hong Kong SAR; 5 Li Ka Shing Institute of Health Sciences, Chinese University of Hong Kong, Shatin, Hong Kong SAR; 6 School of Chemistry and Molecular Biosciences, The University of Queensland, Brisbane, QLD 4072, Australia; 7 Protein Discovery Centre, QIMR Berghofer Medical Research Institute, Brisbane, QLD 4006, Australia; 8 Division of Radiation Oncology, Peter MacCallum Cancer Centre, Melbourne, VIC 3052, Australia; 9 Sir Peter MacCallum Department of Oncology, The University of Melbourne, Melbourne, VIC 3052, Australia; 10 UQ Thoracic Research Centre, The University of Queensland, Brisbane, QLD 4072, Australia; 11 The Prince Charles Hospital, Brisbane, QLD 4032, Australia; 12 Frazer Institute, The University of Queensland, Brisbane, QLD 4102, Australia; 13 Olivia Newton-John Cancer Research Institute, Melbourne, VIC 3084, Australia; 14 School of Cancer Medicine, La Trobe University, Melbourne, VIC 3084, Australia; 15 The University of Queensland Centre for Clinical Research (UQCCR), Brisbane, QLD 4029, Australia; 16 School of Medicine, Queensland University of Technology, Brisbane, QLD 4102, Australia; 17 Department of Surgery, University of Melbourne, Melbourne, VIC 3010, Australia; 18 Division of Urology, Royal Melbourne Hospital, Melbourne, VIC 3052, Australia; 19 Department of Urology, Peninsula Health, Melbourne, VIC 3199, Australia; 20 Sid Faithfull Brain Cancer Laboratory, QIMR Berghofer Medical Research Institute, Brisbane, QLD 4006, Australia; 21 Statistics Group, QIMR Berghofer Medical Research Institute, Brisbane, QLD 4006, Australia; 22 Cancer Control Group, QIMR Berghofer Medical Research Institute, Brisbane, QLD 4006, Australia

**Keywords:** extracellular vesicles, liquid biopsy, multi-cancer early diagnosis, lung cancer screening, miniaturized biosensor, surface-enhanced Raman spectroscopy, microfluidics

## Abstract

Small extracellular vesicles (sEVs) offer a promising, non-invasive method for cancer detection. Despite global research efforts, successful translation of sEV-based diagnostics remains limited. In this study, we identify a 4-protein sEV biomarker panel (thrombospondin-1, nidogen-1, pentraxin-3, and versican) based on proteomic profiles obtained from an isogenic cancer cell line model. The panel’s performance is validated across 22 cancer cell lines and 764 retrospective plasma/serum samples spanning multiple cancer types, yielding robust performance (area under the curve [AUC]: 0.91–1.00). To facilitate clinical application, we develop a multiplex sEV device that integrates nanoshearing-based microfluidics and surface-enhanced Raman scattering (SERS) for simultaneous detection of the 4-protein panel. Using this device on a prospective cohort of 68 patients, we accurately differentiate between benign lung changes and early-stage lung cancer. These findings underscore the potential of sEVs as diagnostic markers for cancer screening. Furthermore, the multiplex microfluidic device’s scalability, simplicity, and cost-effectiveness indicate feasibility for large-scale population screening.

## Highlights

• Protein markers in small extracellular vesicles discovered via an isogenic cell model
• Validation in 22 cell lines and 764 clinical samples across multiple cancer types
• Integration with a microfluidic SERS device allows simultaneous marker readout
• Accurate differentiation of early-stage malignant from benign lung nodules

## Graphical abstract

**Figure:**
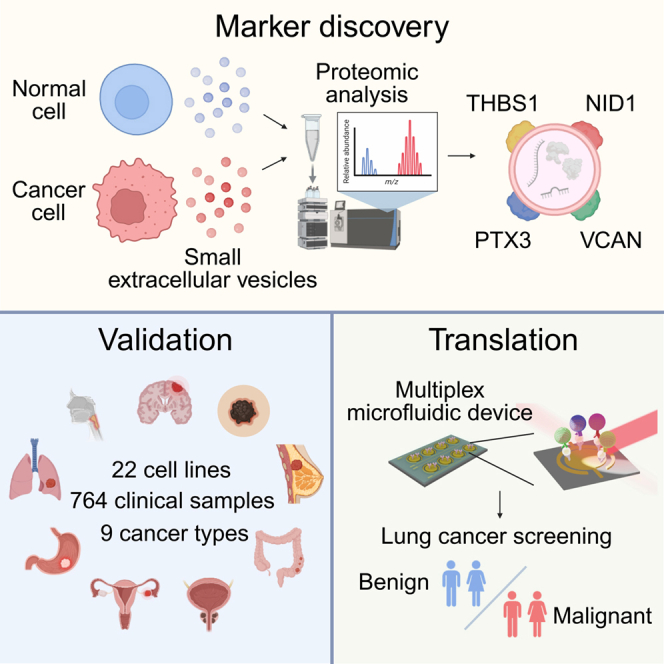

## Teaser

Lobb et al. identify a 4-protein small extracellular vesicle signature for early-stage cancer detection and validate in 22 cell lines and 764 clinical samples spanning 9 cancer types. The signature is integrated with a multiplex microfluidic SERS device that distinguishes malignant from benign lung lesions, enabling scalable, noninvasive screening.

## Introduction

Despite screening and therapeutic advancements, the global cancer burden is steadily rising, with 1 in 5 men and 1 in 6 women developing cancer in their lifetime.^1^ Furthermore, 1 in 8 men and 1 in 10 women will die from untreatable progression of cancer,^1^ making cancer one of the leading causes of death worldwide. Addressing this significant unmet clinical need requires early and accurate cancer detection, which offers the best chance of long-term survival for patients, especially those with early-stage, localized disease.^2^ For this reason, a non-invasive blood test for cancer detection needs to be highly sensitive and specific to be implemented in routine population screening. Various strategies have been explored to analyze human biofluids effectively for cancer detection, including the examination of tumor-secreted factors and circulating tumor DNA.^3^ Recent studies have indicated that a combined approach utilizing both protein and DNA targets, encompassing known mutations and secreted proteins, show promising efficacy in detecting localized cancers and identifying their tissue of origin,^3^ but sensitivity and specificity even in those tests remain hugely problematic.^4^ Therefore, the detection of early-stage disease remains a formidable challenge.

Our study aims to address this diagnostic gap by focusing on the potential of small extracellular vesicles (sEVs) as promising entities for cancer detection. sEVs are membrane-bound vesicles (30–150 nm in diameter) released by all cells, including cancer cells, and are distinguished by their morphology, size, and marker proteins, such as CD9, CD63, CD81, flotillin-1, and TSG101.^5^^,^^6^ Cancer-derived sEVs have been demonstrated to act at various stages of cancer progression and are generally thought to promote cancer growth and metastasis.^5^^,^^7^^,^^8^^,^^9^^,^^10^^,^^11^ The complex cargo of sEVs allows for multicomponent diagnostic analysis that could potentially improve diagnostic sensitivity and specificity.^5^ The protein content of sEVs is dependent on the cell of origin,^12^^,^^13^ and it is now emerging that sEVs represent a viable source of material for diagnostic and prognostic purposes.^5^^,^^14^ Identifying cancer-specific sEV markers could enable the identification of patients with cancer and potentially result in improved survival rates.

While sEVs hold promise for cancer detection, addressing challenges such as rarity, heterogeneity, and non-specificity for their reliable analysis is crucial for translating this potential into clinical practice. Herein, using an isogenic cancer model, we discovered a 4-protein sEV biomarker panel associated with oncogenic transformation. The clinical performance of this 4-protein sEV biomarker panel was validated in cell lines and a large retrospective patient cohort comprising patients diagnosed with multiple malignancies, showing that the biomarker panel was highly sensitive and specific and able to detect stage I disease. Subsequently, to facilitate translation of this bench-based research to the bedside, we developed a multiplex microfluidic device for accurate detection of the 4-protein sEV biomarker panel in a true, prospective cancer screening context, thereby providing major opportunities to significantly improve survival outcomes for cancer patients.

## Results

### Principle of establishing an sEV biomarker panel for early-stage cancer detection

**Figure 1:**
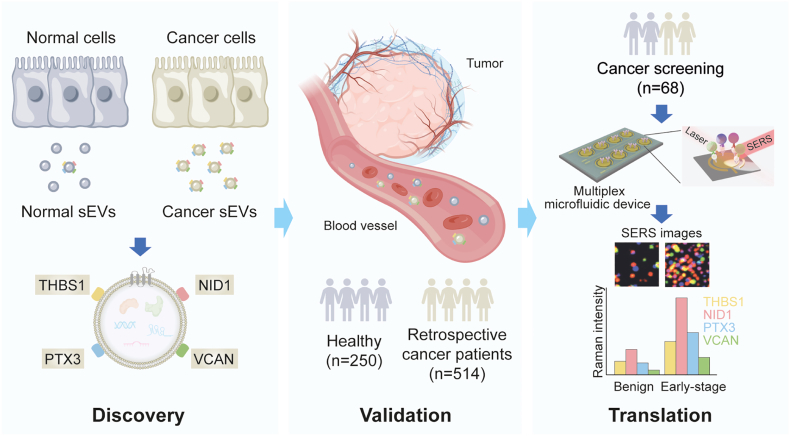
Graphical summary of the study In the *Discovery* phase, a 4-protein biomarker panel (THBS1, NID1, PTX3, and VCAN) was discovered by proteomic analysis of sEVs derived from an isogenic HBEC model using mass spectrometry. The biomarker panel was tested in sEVs isolated from 22 cancer cell lines by ELISA. In the *Validation* phase, a cohort consisting of 250 healthy individuals and 514 patients with multiple cancers was recruited to assess the performance of the biomarker panel. Plasma/serum sEVs were isolated and analyzed by ELISA. In the *Translation* phase, a multiplex microfluidic device incorporating SERS was developed for simultaneous profiling of the 4-protein biomarker panel in a lung cancer screening setting. The expression levels of 4 proteins are reflected by the Raman intensities of the corresponding Raman reporters.

The principle of establishing an sEV biomarker panel for early-stage cancer detection is demonstrated in Figure 1. In the *Discovery* phase, a 4-protein biomarker panel (thrombospondin-1 [THBS1], nidogen-1 [NID1], pentraxin-3 [PTX3], and versican [VCAN]) contained in sEVs was discovered using an isogenic cancer cell line model of human bronchial epithelial cells (HBECs). The biomarker panel was then tested in sEVs derived from 22 cancer cell lines, including non-small cell lung cancer (NSCLC), glioblastoma, colorectal cancer, breast cancer, prostate cancer, melanoma, esophageal cancer, and ovarian cancer. In the *Validation* phase, 764 blood samples from healthy subjects and cancer patients, spanning 8 cancer types, including NSCLC, glioblastoma, colorectal cancer, prostate cancer, melanoma, gastric cancer, esophageal cancer, and small cell lung cancer, were tested for the 4-protein sEV biomarker panel. The presence and abundance of the sEV biomarker accurately discriminated healthy subjects from cancer patients, even those with stage I disease. In the *Translation* phase, a multiplex microfluidic device was developed, capable of simultaneous profiling of the 4-protein sEV biomarker. This device incorporated an alternating current electro-hydrodynamically-induced nanoshearing strategy and surface-enhanced Raman scattering (SERS) nanotag barcodes to enhance the specificity and accuracy of cancer detection. Our device was able to accurately discriminate benign from malignant lung changes in a prospectively collected patient cohort.

### Development of protein diagnostic signature from sEVs isolated from an isogenic cell line

**Figure 2:**
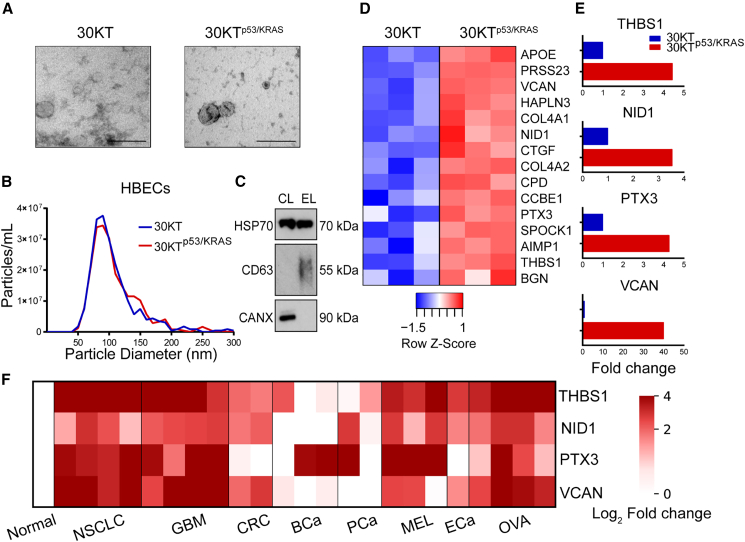
Transformation-induced changes to the protein composition of cell-derived sEVs (A) The morphology of isolated sEVs was assessed using transmission electron microscopy. Images of normal and transformed HBEC-derived sEVs (scale bars, 200 nm). (B) Nanoparticle analysis using tunable resistive pulse sensing of sEVs isolated from HBECs demonstrates that the majority of sEVs have a size range between 30 and 150 nm, and that transformation does not result in an increase in sEV secretion. (C) Western blot of sEVs from HBECs demonstrating the presence of sEV proteins HSP70 and CD63 and the absence of the cell marker calnexin. (D) Label-free mass spectrometry identified 148 proteins with greater abundance in sEVs derived from transformed HBECs (FDR <0.02), of which 15 were annotated as extracellular proteins. (E) Mass spectrometry results were confirmed using ELISA for THBS1, NID1, PTX3, and VCAN in sEVs derived from normal and transformed HBECs. (F) sEVs derived from 22 cancer cell lines including NSCLC (SKMES1, H1650, HCC4006, and H2170), glioblastoma ([GBM], D54, D270, U87, and U118), colorectal cancer ([CRC], HT29 and SW620), breast cancer ([BCa], BT549, MDA231, and MDA436), prostate cancer ([PCa], PC3 and LNCaP), melanoma ([MEL], A375, MAMEL65, and SKMEL28), esophageal cancer ([ECa], OE19), and ovarian cancer ([OVA], A2780, CAOV3, IGROV1, and OVCAR8) showed a clear increase in expression of THBS1, NID1, PTX3, and VCAN in relation to the average levels of sEVs from normal cells ([HBEC] 30KT, HOSE 6.3, and HOSE 17.1). Samples in mass spectrometry and ELISA were measured in triplicate. See also Tables S1 and S2.

In order to develop a cancer-specific protein diagnostic signature, we hypothesized that the protein content of sEVs derived from cancer cells would be sufficiently different from that of sEVs derived from untransformed cells and that these differences could form the basis of a diagnostic assay. Specifically, we postulated that an isogenic model of HBECs transformed by downregulation of p53 and overexpression of KRAS (30KT^p53/KRAS^) would secrete sEVs with a distinct protein profile. We isolated sEVs secreted by untransformed (30KT) and transformed (30KT^p53/KRAS^) HBECs. Transmission electron microscopy (TEM) and nanoparticle analysis demonstrated that both sets of sEVs displayed typical size distribution and morphology (Figures 2A and 2B), and immunoblotting indicated the presence of canonical sEV markers (Figure 2C).

The proteomes of normal and transformed HBECs were then evaluated using mass spectrometry. Label-free quantification indicated the presence of sEV markers Alix, CD9, CD63, CD81, and TSG101 in both sets of sEVs (Table S1). Further statistical analysis revealed 327 differentially abundant proteins (false discovery rate [FDR] <0.02) between untransformed and transformed sEVs (Table S2). Of the 327 differentially abundant proteins, 15 were annotated as extracellular proteins that could potentially be utilized in rapid clinical assays (Figure 2D). From these 15 proteins, we selected 4 targets (THBS1, NID1, PTX3, and VCAN) based on their established roles in tumor progression including extracellular matrix remodeling, immune modulation, and stromal signaling.^15^^,^^16^^,^^17^^,^^18^^,^^19^^,^^20^ Additionally, these 4 targets have been identified on the surface of intact sEVs in multiple cancer contexts,^21^^,^^22^^,^^23^^,^^24^ which facilitates direct antibody-based detection without requiring complex lysis or proteomic workflows, enhancing assay simplicity and compatibility with point-of-care formats. Confirmed by ELISA assays, these 4 proteins were elevated in transformed HBEC-derived sEVs (Figure 2E).

### Assessment of THBS1, NID1, PTX3, and VCAN in sEVs from patients of different cancer types

We next sought to determine the general diagnostic capability of this 4-protein sEV biomarker panel. First, we evaluated the expression level of these proteins in sEVs derived from 22 cancer cell lines, comprising NSCLC, glioblastoma (GBM), colorectal (CRC), breast (BCa), prostate (PCa), melanoma (MEL), esophageal (ECa), and ovarian (OVA) cancers (Figure 2F). Strikingly, we found that all 4 proteins were generally upregulated in all cancer cell line-derived sEVs compared to levels from non-tumor cells, regardless of the cancer type (Figure 2F). These findings suggested that this 4-protein sEV biomarker panel could potentially be used to diagnose multiple human cancer types.

To investigate this, we isolated sEVs from the serum/plasma of 250 healthy, gender- and age-matched control subjects and 514 cancer patients, who had been diagnosed with cancers of the lung, brain, colorectum, prostate, melanoma, stomach, or esophagus. The median age of both healthy controls and patients at diagnosis was 65.5 (range 21–90; Table S3). TEM, nanoparticle, and immunoblotting analysis of sEVs isolated from healthy individuals and cancer patients revealed lipid bilayer vesicles with an average diameter between 30 and 150 nm and expression of CD9 and flotillin-1 (Figure S1).

**Figure 3:**
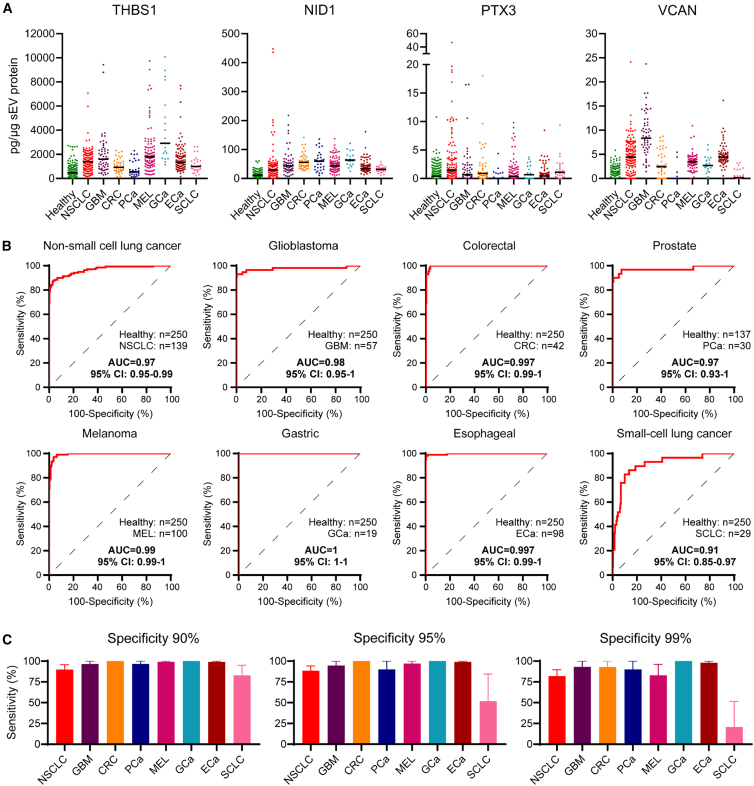
The transformed sEV signature accurately diagnoses cancer in patient plasma (A) The expression levels of THBS1, NID1, PTX3, and VCAN in plasma derived from cancer patients are increased in comparison to healthy controls. Samples were measured in triplicate. Lines in dot plots represent median values. (B) ROC curves of classification of each cancer type compared to healthy controls demonstrate excellent diagnostic capability of the 4-protein sEV biomarker panel with an AUC of 0.91–1. (C) The sensitivity of the diagnostic sEV signature for each cancer type was evaluated at a fixed specificity of 90%, 95%, and 99%. Error bars represent 95% confidence intervals. Healthy (*n* = 250), NSCLC (*n* = 139), glioblastoma ([GBM], *n* = 57), colorectal cancer ([CRC], *n* = 42), prostate cancer ([PCa], *n* = 30), melanoma ([MEL], *n* = 100), gastric cancer ([GCa], *n* = 19), esophageal cancer ([ECa], *n* = 98), small cell lung cancer ([SCLC], *n* = 29). See also Figures S1–S5 and Table S3.

We found that all 4 signature proteins, THBS1, NID1, PTX3, and VCAN, were significantly increased in sEVs derived from cancer patients compared to healthy controls (Figure 3A). Each protein from the sEV signature had a range of diagnostic capabilities in the different cancer cohorts, as assessed by receiver operating characteristic (ROC) curves (Figures S2–S5). Furthermore, logistic regression analysis utilizing a combination of age, gender, and signature proteins was able to generate a highly significant separation of healthy controls and cancer patients, with an area under the curve (AUC) of 0.91–1 (Figure 3B).

At fixed specificity levels of 90%, 95%, and 99%, the median sensitivity of the diagnostic sEV signature across all 8 cancer types was 92.1% (95% confidence interval [CI]: 89.4%, 94.2%), 89.0% (95% CI: 85.9%, 91.5%), and 80.1% (95% CI: 76.3%, 83.4%), respectively. This ranged from 75.9% (SCLC) to 100% (GCa) at 90% specificity, 51.7% (SCLC) to 100% (GCa) at 95% specificity, and 20.7% (SCLC) to 100% (GCa) at 99% specificity (Figure 3C), indicating that the sEV signature has excellent diagnostic capacity.

**Figure 4:**
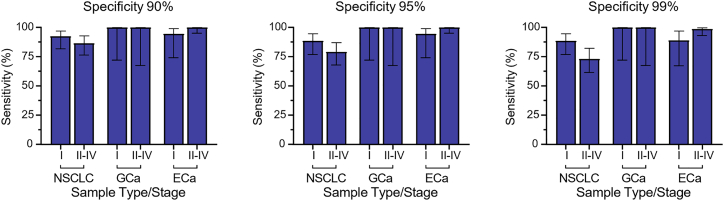
Sensitivity of the diagnostic sEV signature according to tumor stages The diagnostic sEV signature is capable of diagnosing early-stage disease (stage I) as well as later-stage disease (stage II-IV) at 90%, 95%, and 99% specificities in NSCLC (stage I, *n* = 52; stage II-IV, *n* = 67), GCa (stage I, *n* = 10; stage II-IV, *n* = 8), and ECa (stage I, *n* = 18; stage II-IV, *n* = 77). Error bars represent 95% CI.

For a liquid biopsy to be of maximal benefit, it needs to identify patients at the earliest possible stage to enable the best opportunity for intervention. We, therefore, next assessed the capability of the diagnostic sEV signature to identify early-stage disease. For this, we were able to evaluate the sensitivity of the 4-protein sEV biomarker panel in stage I versus stage II–IV disease in NSCLC and esophageal and gastric cancer. Remarkably, the sensitivity for detecting stage 1 disease at 90%, 95%, and 99% specificity was comparable to the sensitivity for detecting later-stage disease in all 3 cancer types (Figure 4), demonstrating that the diagnostic sEV signature is highly efficient at detecting early-stage tumors.

### Development of a scalable multiplex microfluidic device for cancer screening

**Figure 5:**
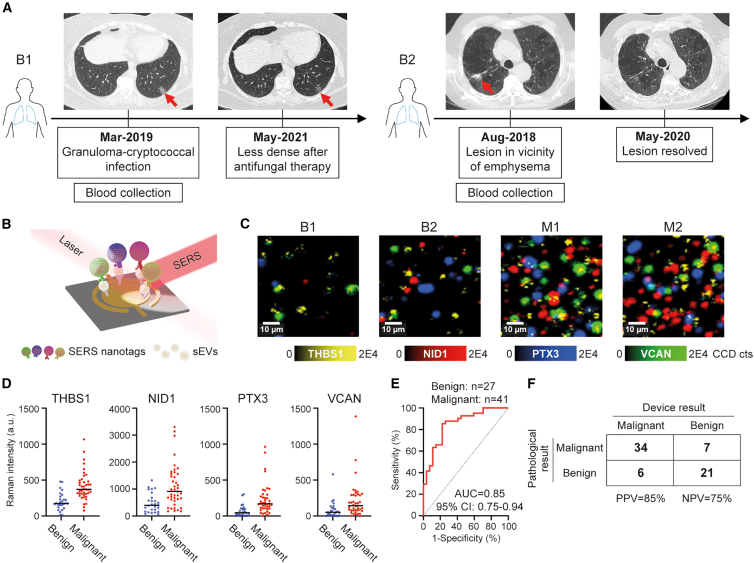
Evaluation of a multiplex microfluidic device applicable for liquid biopsy testing in a cancer screening setting (A) Clinical follow-up by CT imaging of 2 representative benign patients B1 and B2. Red arrows indicated nodules in patients’ lungs. B1 had a granuloma-cryptococcal infection, and the lesion was found less dense after 26 months. B2 had a lesion in the vicinity of emphysema, which resolved after 21 months. (B) Schematic of multiplex microfluidic device consisting of a pair of asymmetric circular electrodes. Electrodes are conjugated with an anti-THBS1 antibody to capture cancer-derived sEVs. SERS nanotags carrying designated Raman reporters and paired target antibodies (against THBS1, NID1, PTX3, and VCAN) are used for labeling captured sEVs and then analyzed by SERS mapping. (C) Representative false-color SERS spectral images demonstrating an enrichment of THBS1, NID1, PTX3, and VCAN in early-stage NSCLC patients (M1 and M2) compared to patients with benign diseases (B1 and B2). Scale bars, 10 μm. (D) The Raman intensity of each biomarker THBS1, NID1, PTX3, and VCAN in benign (*n* = 27) and early-stage NSCLC (*n* = 41) patients. a.u., arbitrary units. Samples were measured in triplicate. Lines in dot plots represent median values. (E) ROC curve of logistic regression classification indicating an AUC of 0.85 in detecting early-stage NSCLC cases compared to benign cases in a cancer screening setting. (F) The confusion matrix of the multiplex microfluidic device. See also Figures S11 and S12 and Table S4.

With promising results observed across a diverse pan-cancer cohort, we next embarked on leveraging nanotechnologies to further elevate the clinical performance of this diagnostic for early-stage cancer screening. Our goal was to integrate the 4-protein sEV biomarker panel with state-of-the-art nanotechnologies for precise, easy-to-use cancer diagnosis. Owing to their advantageous features for analyzing liquid biopsies, we opted to use a multiplex microfluidic device with an integrated nanoparticle barcode system to develop a sandwich immunoassay for specific on-chip sEV capture and detection (Figures 5B and S6). The unequal charge density on the larger and smaller circular electrodes (Figure S6) generates shear forces and a “nanoshearing” effect that increases sEV collisions with the immobilized capture antibodies providing improved specificity and sensitivity.^25^ 60-nm gold nanoparticles conjugated with different pairs of Raman reporters and antibodies targeting the sEV signature proteins (Figure S7) were subsequently used to barcode the surface proteins of captured cancer-derived sEVs. This approach combines the improved specificity provided by the nanoshearing phenomenon with the increased sensitivity resulting from the enhanced Raman reporter signal due to the strong plasmonic effect of gold nanoparticles, enabling highly precise monitoring of cancer sEVs.

To ensure our capture and readout is specific for cancer sEVs, purified plasma sEVs were captured on functionalized microelectrodes in the presence of an alternating current electric field. The sEV protein concentrations were quantified using the Micro BCA assay, and 0.6 μg of total sEV protein was loaded onto the detection device for each sample. Microelectrodes were conjugated with anti-THBS1 antibody to capture and enrich circulating cancer-derived sEVs as THBS1 exhibited the highest expression level among the 4 markers (Figure 3A) and provided clear distinction between healthy individuals and NSCLC patients compared to the anti-NID1 antibody for capture (Figure S8).

Next, the specificity of the multiplex microfluidic device was verified using H1975 NSCLC-derived sEVs (Figure S9). In retrospective NSCLC samples used above in ELISA-based testing, the 4-protein sEV biomarker was significantly elevated in late-stage NSCLC patients (*n* = 6) compared to healthy controls (*n* = 4) using the multiplex microfluidic device (Figures S10A and S10B), demonstrating the platform-independent robustness of the biomarker. The combination of 4 markers showed a perfect classification performance with an AUC of 1 (Figure S10C). This combined strategy of nanoshearing and SERS, which enabled improved capture efficiency of sEVs and highly sensitive multiplex *in situ* readout, allowed for the accurate identification of the diagnostic sEV signature in liquid biopsies.

### Translation into a cancer screening setting

Up until this point, we limited our analysis to individuals with confirmed cancer and controls from healthy individuals. This, however, fails to reflect the complex dynamic that is present in a true cancer screening setting. Many individuals there may have inflammatory conditions and/or other benign diseases that could potentially increase the false-positive rate. For this reason, we applied our highly sensitive multiplex microfluidic device to patients who are suspected to having lung cancer from lesions detected on lung computed tomography (CT) scans in a prospective clinical study (ACTRN12618001789257). These patients then undergo a biopsy of the suspected lesions in the thoracic ward of a tertiary hospital. Malignant diagnoses were based on the biopsy results. For benign diagnoses, biopsies were also used when pathology was positive for a specific diagnosis. If the biopsy results were non-specific for benign diseases, patients would receive repeat CT scans to demonstrate either improvement or stability of the lesion over 12 months of follow-up (Figure 5A). Clinical information of these patients is indicated in Table S4. We postulated that the multiplex microfluidic device would be capable of detecting malignant lesions and streamline the diagnostic process as well as preventing benign patients from undergoing unnecessary invasive biopsies.

We then evaluated the 4-protein sEV biomarker abundance in purified sEV samples from a cohort of 68 indiscriminately selected patients to ensure unbiased representation across the sample population. After clinical follow-up, a total of 27 patients were determined to have benign lung diseases and 41 patients to have early-stage lung cancer. Purified sEVs from representative benign and malignant patients were characterized by the expression of sEV proteins as well as particle amounts (Figures S11A and S11B). Moreover, purified sEVs from representative benign and malignant patients were loaded onto the electrode functionalized with anti-THBS1 antibody and labeled by SERS nanotags against CD9 and CD63, confirming that sEVs were successfully captured on the device (Figures S11C and S11D). Compared to patients with benign lung diseases, patients with early-stage lung cancer showed an increase of all 4 biomarkers in malignant samples (Figures 5C and 5D). ROC curves of each biomarker were generated, with AUC values ranging from 0.75 to 0.84 (Figure S12). We then assessed the diagnostic sensitivity and specificity of the combined 4-protein sEV biomarker, and obtained a classifier of malignant and benign lesions with an AUC of 0.85 (Figure 5E), with a positive predictive value of 85% and a negative predictive value of 75% (Figure 5F). These findings demonstrated that our multiplex microfluidic device is capable of identifying cancer patients with a high degree of accuracy using a simple liquid biopsy.

### Validation of the relationship between the protein diagnostic signature and tumor presence

**Figure 6:**
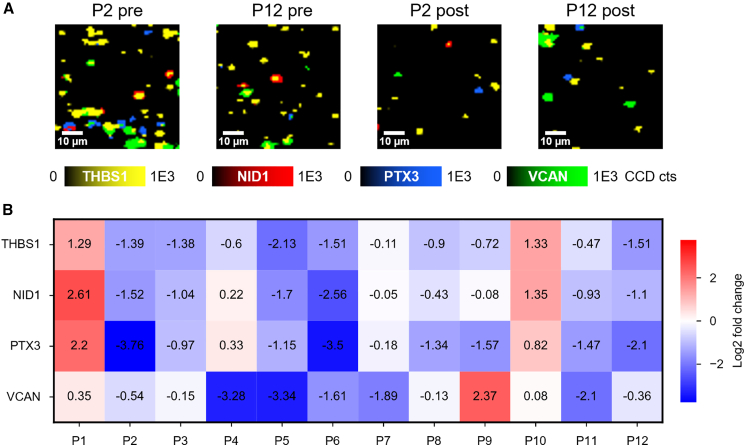
Evaluation of the multiplex microfluidic device in a longitudinally monitored cohort of pre- and post-surgery NSCLC patients (A) Representative false-color SERS spectral images demonstrating a decrease of THBS1, NID1, PTX3, and VCAN in post-surgery NSCLC patients (P2 post and P12 post) compared to paired pre-surgery (P2 pre and P12 pre) patients. Scale bars, 10 μm. (B) Heatmap showing the log_2_ fold changes in Raman intensities of THBS1, NID1, PTX3, and VCAN in post-surgery NSCLC patients (*n* = 12), relative to their paired pre-surgery samples. Samples were measured in triplicate. Negative values (blue) indicate decreased expression after surgery, while positive values (red) indicate increased expression. See also Table S5.

To confirm the relationship between the 4-protein sEV biomarker panel and tumor presence, we performed a longitudinal analysis using paired pre- and post-surgery plasma samples from 12 NSCLC patients. sEVs were isolated from each sample and analyzed using the multiplex microfluidic device to quantify the expression levels of THBS1, NID1, PTX3, and VCAN. The clinical characteristics of the cohort are provided in Table S5. Although few outliers were observed, the overall trend of reduced marker expression following surgical removal of the tumor suggested a potential relationship between the sEV protein signature and tumor burden (Figure 6). These outliers may reflect inter-patient heterogeneity in tumor biology, incomplete resection, or early signs of recurrence and warrant further investigation.

## Discussion

Considerable effort has been devoted to exploring blood-based markers for cancer diagnosis and prognosis. Initial endeavors focused on mass spectrometry-based identification of cancer-specific protein markers have been hampered by challenges in the dynamic range of the plasma proteome.^26^ Importantly, when it comes to identifying circulating protein biomarkers, analysis of sEVs negates these issues. In complex biological fluids, like blood, the expression of proteins can span several orders of magnitude.^27^ Given that the total purified sEV protein makes up a small proportion of total plasma protein (sEV protein <300 μg/mL,^28^ compared to >50 mg/L plasma protein^29^), this mitigates dynamic range issues.^30^

The concept that sEVs can be utilized as biomarkers for diagnostic or prognostic/diagnostic purposes in cancer has received considerable recent attention.^9^^,^^10^^,^^11^^,^^31^ While individual biomarkers like glypican-1 have shown promise in diagnosing pancreatic cancer with high specificity and sensitivity,^31^ the complexity of cancer necessitates a multi-faceted approach. This is exemplified by the CancerSEEK and GRAIL tests, which integrate various biomarkers and technologies for improved cancer detection.^3^^,^^32^ Based on the promising results of CancerSEEK, a prospective study called DETECT-A was conducted.^33^ A total of 10,006 women with no prior history of cancer were evaluated with the CancerSEEK platform, coupled with positron emission tomography-CT imaging.^33^ During the study period, 26 (27%) women with cancer were detected by CancerSEEK, including 5 at stage I (19%), 3 at stage II (12%), 8 at stage III (31%), and 9 at stage IV (35%).^33^ However, 24 (25%) additional cancers were detected by standard-of-care screening, while 46 (48%) were not detected by either approach.^33^ Furthermore, although GRAIL has made significant advancements in the detection of cancer, this test is still very limited in early-stage cancer with a sensitivity of 39%.^32^ These results indicate that both GRAIL and CancerSEEK are still limited in early-stage detection and clinical utility.^34^ In comparison, our study focuses on harnessing sEVs and introduces a robust multi-protein signature, providing a compelling alternative to existing liquid biopsy methods for early-stage cancer screening.

Complementary to our findings here, a report on the proteome content of cancer sEVs also identified multi-protein signatures capable of cancer detection.^35^ Interestingly, a combination of 3 EV proteins, including VCAN, which we have identified, was capable of classifying tumors and normal with a sensitivity of 90% and a specificity of 94%. Moreover, a machine learning approach using the identified EV cargo resulted in a sensitivity of 95% and a specificity of 94% in classifying cancer.^35^ While large numbers of samples were analyzed, a detailed confirmation or development of a specific diagnostic application, as we have done in this study, was not performed. Importantly, while our test found some overlapping biomarkers, we were uniquely capable of early-stage cancer detection, further expanding the translational applicability of sEV biomarkers in cancer screening.

The 4 selected markers contribute to the tumor progression through distinct yet complementary pathways: THBS1 modulates angiogenesis and immune evasion via CD47 signaling,^36^ NID1 facilitates pre-metastatic niche formation by promoting extracellular matrix remodeling,^18^ PTX3 is involved in inflammation and immune cell recruitment,^37^ and VCAN supports tumor invasiveness and mesenchymal transition.^38^ The presence of these proteins in sEVs suggests a functional link between tumor cell behavior and sEV-mediated intercellular communication. It is likely that the diagnostic signal we observed stems from the dynamic release of these surface proteins as part of tumor-driven sEV secretion. Our study used p53 downregulation and KRAS overexpression to induce transformation in HBECs, which served as a model for developing the sEV protein signature. Importantly, our *in vitro* validations across 22 cancer cell lines indicated that the enrichment of THBS1, NID1, PTX3, and VCAN in sEVs is not confined to KRAS-driven cancers. Instead, similar upregulation of these 4 markers is observed in sEVs derived from tumor cells harboring various oncogenic mutations, suggesting that the alterations in the sEV proteome represent a more generalizable feature of malignant transformation, rather than being solely dependent on specific oncogenic drivers. By targeting this subset of tumor-associated, functionally active sEVs, our platform captures clinically informative signals directly related to underlying tumor biology. The remaining 11 candidates (Figure 2D), although not included in the final diagnostic panel, also possess notable biological relevance. They are broadly involved in key cancer-associated processes such as extracellular matrix remodeling, angiogenesis, immune modulation, and tumor cell migration. Collectively, they contribute to tumor progression by shaping the tumor microenvironment, promoting metastatic potential, and supporting stromal activation.^39^^,^^40^^,^^41^^,^^42^^,^^43^^,^^44^^,^^45^^,^^46^^,^^47^^,^^48^ While these proteins were not included in our final panel, they remain attractive candidates for further investigation and expansion in tumor sEVs.

In addition to analyzing the sEV proteome, sEV RNA has gained great attention as biomarkers for cancer diagnosis^49^ and has been approved by the FDA for testing in prostate cancer.^50^^,^^51^^,^^52^^,^^53^ In other examples, changes of levels in non-coding RNA including microRNA, long non-coding RNA and circular RNA in EVs have been found in breast cancer,^54^^,^^55^^,^^56^^,^^57^ prostate cancer,^58^^,^^59^^,^^60^ lung cancer,^61^^,^^62^^,^^63^ and other cancers.^64^^,^^65^^,^^66^^,^^67^ However, these studies have yet to be commercialized for early-stage testing.

Despite their promise, sEV-based diagnostics face translation barriers, including inconsistent isolation/characterization standards, which are now being gradually addressed through community-wide efforts,^6^ and limited commercialization pathways. Our platform addresses these gaps by enabling multiplexed detection of a 4-protein panel directly on intact sEVs, improving sensitivity, specificity, and subpopulation targeting without the need for lysis or amplification. This streamlined workflow preserves vesicle integrity and is validated across isogenic models, diverse cancer cell lines, and independent patient cohorts. Compared with invasive or low-sensitivity modalities such as biopsies or imaging, our method offers repeatable, non-invasive sampling for longitudinal monitoring, and its integration of nanoshearing microfluidics with SERS provides a scalable foundation for future portable, point-of-care applications. By enabling the simultaneous detection of multiple cancers from a single device, our biomarker signature and technology platform have the potential for improving early detection, potentially leading to better patient outcomes, thereby encapsulating the steps from bench-based research to bedside diagnostic technology establishment.

Future directions involve recruiting a diverse cohort of symptomatic patients prior to traditional diagnostic testing, providing an unbiased measure of the platform’s sensitivity and specificity in real-world conditions. Additionally, longitudinal studies could be designed to assess the platform’s effectiveness in detecting cancer at early, asymptomatic stages or to explore its prognostic utility, thereby further strengthening its value for routine screening and disease monitoring. Our current readout requires a confocal Raman spectrometer, which limits accessibility. Future integration with portable, user-friendly Raman devices will improve deployment. Although presently limited to 4-plex detection, the use of spectrally distinct SERS nanotags allows theoretical expansion to ultra-multiplex formats (up to 26-plex),^68^ enabling broader sEV phenotyping for future diagnostic and monitoring applications. Moreover, future single-EV studies will be important to definitively distinguish marker-specific enrichment from potential abundance contributions.

### Limitations of the study

First, diagnostic performance varied across tumor types, indicating that additional or tumor-specific markers may be required for refined cancer classification. Second, the prospective study was conducted at a single center, focused on lung cancer, and involved a modest sample size, which may constrain generalizability. Third, the SERS-based average readout is semi-quantitative and measures bulk sEV populations enriched by immunocapture, without resolving single-vesicle heterogeneity. Finally, only blood-derived sEVs were evaluated; for some cancers such as prostate cancer, alternative biofluids (e.g., urine) may provide superior diagnostic performance and should be explored in future studies.

## Resource availability

### Lead contact

Further information and requests for resources and reagents should be directed to and will be fulfilled by the lead contact, Andreas Möller (andreas.moeller@ent.cuhk.edu.hk).

### Materials availability

This study did not generate new unique reagents.

### Data and code availability

• The mass spectrometry proteomics data have been deposited to the ProteomeXchange Consortium via the PRIDE^69^ partner repository with the dataset identifier PXD073635 and are publicly available as of the date of publication. Processed proteomics data are available in Table S2.
• This paper does not report original code.
• Any additional information required to reanalyze the data reported in this paper is available from the lead contact upon request.

## Acknowledgments

The authors would like to thank Professor Rajiv Khanna, QIMR Berghofer, for assistance with the project and sample identifications and the members of the Tumour Microenvironment Laboratory of QIMR Berghofer Medical Research Institute and the Centre for Personalized Nanomedicine at AIBN for critical input, discussions, and proofreading of the manuscript. This work was supported by the 10.13039/501100000925National Health and Medical Research Council grant to A.M. and M.T. (APP1185907), to A.M. and K.M.F. (APP1164020), to R.J.L. (2034433), and to A.W. (2034488). The 10.13039/501100004853Chinese University of Hong Kong provided funding for this work to A.M. and K.S.V. (IDBF23MED14). A.M. was supported by the 10.13039/501100003452Innovation and Technology Commission, Hong Kong SAR (PiH/048-050/22GS), the Global STEM scheme (GSP153), and the Hong Kong Jockey Club Charities Trust. M.T. acknowledges funding from the 10.13039/501100000923Australian Research Council (FL220100059). M.T., R.J.L., A.W., and A.M. acknowledge funding from 10.13039/501100001111Cancer Australia (2010799). The authors thank the Australian National Fabrication Facility of Queensland Node (ANFF-Q) for providing instruments for biosensor fabrication and SERS mapping. The authors acknowledge the facilities, and the scientific and technical assistance, of the Australian Microscopy & Microanalysis Research Facility at the Center for Microscopy and Microanalysis, The University of Queensland. Figure 1 was created in BioRender (Zhou Q. [2026] https://biorender.com/embrhrt) and Servier Medical Art (https://smart.servier.com/), licensed under CC BY 4.0 (https://creativecommons.org/licenses/by/4.0/). The graphical abstract was created in BioRender (Zhou Q., [2026] https://biorender.com/obg3xur).

## Author contributions

Conceptualization, R.J.L., M.T., A.M.; design and supervision of experiments, R.J.L., M.T., and A.M.; conducting experiments, R.J.L., Q.Z., A.W., J.W., and K.S.V.; design and supervision of mass spectrometry, J.J.G.; conducting mass spectrometry and analysis, M.L.H. and E.L.N.; contribution of clinical samples and data, S.E., B.P., L.G.A., V.F.B., E.N., J.M., R.V.B., I.A.Y., N.M.C., C.M.H., M.M.M., B.W.D., D.C.W., N.T., J.M.M., D.F., K.M.F., and A.P.B.; statistical analysis, G.H.; funding acquisition, R.J.L., K.M.F., K.S.V., A.W., M.T., and A.M.; writing – original draft, R.J.L., Q.Z., M.T., and A.M.; writing – reviewing & editing, R.J.L., Q.Z., A.W., J.W., K.S.V., E.L.N., M.L.H., S.E., B.P., L.G.A., V.F.B., E.N., J.M., R.V.B., I.A.Y., N.M.C., C.M.H., M.M.M., J.J.G., B.W.D., G.H., D.C.W., N.T., J.M.M., D.F., K.M.F., A.P.B., M.T., and A.M.

## Declaration of interests

A.M. and R.J.L. have filed a patent (# WO2021077181A1) on the findings presented in this publication. M.T. has patent #US10156546B2 issued to University of Queensland.

## STAR★Methods

### Key resources table

**Table:** 

REAGENT or RESOURCE	SOURCE	IDENTIFIER
**Antibodies**		

Rabbit anti-human calnexin (Clone C5C9)	Cell Signaling Technology	Cat# 2679; RRID: AB_2228381
Rabbit anti-human CD9 (Clone EPR2949)	Abcam	Cat# ab92726; RRID: AB_10561589
Mouse anti-human CD63 (Clone MEM-259)	Abcam	Cat# ab8219; RRID: AB_306364
Mouse anti-human HSP70 (Clone 7)	BD Biosciences	Cat# 610608; RRID: AB_397942
Goat anti-Rabbit IgG (H + L) Secondary Antibody, HRP	Invitrogen	Cat# 31460; RRID: AB_228341
Goat anti-Mouse IgG (H + L) Secondary Antibody, HRP	Invitrogen	Cat# 31430; RRID: AB_228307
Mouse anti-human Flotillin-1 (Clone 18)	BD Biosciences	Cat# 610821; RRID: AB_398140
Rabbit anti-human CD9 (Clone D8O1A)	Cell Signaling Technology	Cat# 13174; RRID: AB_2798139
Mouse anti-human Alix (Clone 1A12)	Santa Cruz Biotechnology	Cat# sc-53540; RRID: AB_673819
Goat anti-Rabbit IgG (H + L) Secondary Antibody, HRP	Cell Signaling Technology	Cat# 7074; RRID: AB_2099233
Horse anti-Mouse IgG (H + L) Secondary Antibody, HRP	Cell Signaling Technology	Cat# 7076; RRID: AB_330924
Mouse anti-human CD9 (Clone MEM-61)	Novus Biologicals	Cat# NB500-327; RRID: AB_10002860
Mouse anti-human CD63 (Clone H5C6)	Novus Biologicals	Cat# NBP2-42225; RRID: AB_2884028
Mouse anti-human THBS1 (Clone 301221)	R&D Systems	Cat# MAB3074; RRID: AB_2255854
Mouse anti-human NID1 (Clone 302117)	R&D Systems	Cat# MAB2570; RRID: AB_2153270
Mouse anti-human PTX3 (Clone 247911)	R&D Systems	Cat# MAB1826; RRID: AB_2173564
Rat anti-human VCAN (Clone 255915)	R&D Systems	Cat# MAB3054; RRID: AB_2241502

**Chemicals, peptides, and recombinant proteins**		

2-Mercaptoethanol	Bio-Rad	Cat# 1610710; CAS: 60-24-2
2-propanol	Supelco	Cat# 109634; CAS: 67-63-0
30% Acrylamide/Bis Solution	Bio-Rad	Cat# 1610156
4x Laemmli Sample Buffer	Bio-Rad	Cat# 1610747
Acetonitrile	Sigma-Aldrich	Cat# 900667; CAS: 75-05-8
Ammonium bicarbonate	Sigma-Aldrich	Cat# 09830; CAS: 1066-33-7
Ammonium Persulfate (APS)	Bio-Rad	Cat# 1610700; CAS: 7727-54-0
AZ 100 Remover	Microchemicals GmbH	Cat# 1000100
AZ 726 MIF Developer	Microchemicals GmbH	Cat# 1000726
AZ nLOF 2020 photoresist	Microchemicals GmbH	Cat# 1A20200100
Bovine pituitary extract	Gibco	Cat# 13028014
Bovine serum albumin	Sigma-Aldrich	Cat# A7030; CAS: 9048-46-8
Dithiobis(succinimidyl propionate)	Thermo Scientific	Cat# 22585; CAS: 57757-57-0
DMSO	Sigma-Aldrich	Cat# 276855; CAS: 67-68-5
DPBS	Gibco	Cat# 14190144
DTNB	Sigma-Aldrich	Cat# D8130; CAS: 69-78-3
Dulbecco’s Modified Eagle Medium (DMEM)	Gibco	Cat# 11965092
EndoGRO-MV Complete Culture Media Kit	Sigma-Aldrich	Cat# SCME004
Epidermal growth factor (EGF)	Gibco	Cat# 37000015
Ethanol	Supelco	Cat# 8.18760; CAS: 64-17-5
Fetal bovine serum	Gibco	Cat# A5670701
GlutaMAX	Gibco	Cat# 35050061
Glycine	Invitrogen	Cat# 15527013
Gold (III) chloride trihydrate	Sigma-Aldrich	Cat# 520918; CAS: 16961-25-4
Iodoacetamide	Sigma-Aldrich	Cat# A3221; CAS: 144-48-9
Keratinocyte serum free medium (KSFM)	Gibco	Cat# 17005042
MBA	Sigma-Aldrich	Cat# 706329; CAS: 1074-36-8
Methanol	Supelco	Cat# 1.06009; CAS: 67-56-1
MPY	Sigma-Aldrich	Cat# 148202; CAS: 4556-23-4
Non-fat powdered milk	Merck	Cat# 1.15363; CAS: 999999-99-4
PBS tablets	Gibco	Cat# 18912014
Penicillin-Streptomycin	Gibco	Cat# 15140122
Precision Plus Protein™ WesternC™ Blotting Standards	Bio-Rad	Cat# 1610376
Protease inhibitors	Sigma-Aldrich	Cat# P8340
Resolving Gel Buffer for PAGE	Bio-Rad	Cat# 1610798
RPMI 1640 Medium	Gibco	Cat# 11875093
SDS	Bio-Rad	Cat# 1610302; CAS: 151-21-3
Sodium azide	Sigma-Aldrich	Cat# 71289; CAS: 26628-22-8
Stacking Gel Buffer for PAGE	Bio-Rad	Cat# 1610799
TEMED	Bio-Rad	Cat# 1610801; CAS: 110-18-9
TFMBA	TCI America	Cat# T25425G; CAS: 5211-44-9
Trifluoroacetic acid	Thermo Scientific	Cat# 85183; CAS: 76-05-1
Tris	Roche	Cat# 10153265103; CAS: 77-86-1
Tri-Sodium citrate	Ajax Finechem	Cat# AJA467-500G; CAS: 68-04-2
TrypLE™ Express Enzyme (1X), no phenol red	Gibco	Cat# 12604013
Trypsin	New England Biolabs (NEB)	Cat# P8101S
Tween 20	Sigma-Aldrich	Cat# P1379; CAS: 9005-64-5
Western Blotting Detection Reagent	Cytiva	Cat# RPN2235

**Critical commercial assays**		

Human THBS1 DuoSet ELISA	R&D Systems	Cat# DY3074
Human NID1 DuoSet ELISA	R&D Systems	Cat# DY2570
Human PTX3 DuoSet ELISA	R&D Systems	Cat# DY1826
Human VCAN ELISA Kit	Novus Biologicals	Cat# NBP2-75353
Amicon® Ultra-4 10 kDa	Millipore	Cat# UFC8100
Micro BCA™ Protein Assay Kit	Thermo Scientific	Cat# 23235
SYLGARD™ 184 Silicone Elastomer Kit	Dow	Cat# 761036
qEVoriginal 70nm SEC columns	IZON Science	Cat# ICO-70

**Deposited data**		

Mass spectrometry proteomics data	This paper	ProteomeXchange Consortium: PXD073635

**Experimental models: Cell lines**		

HBECs, 30KT	Larsen et al.^70^^,^^71^	N/A
30KT^p53/KRAS^	Larsen et al.^70^^,^^71^	N/A
HOSE 6-3	Garvan Research Institute	RRID: CVCL_7673
HOSE 17-1	Garvan Research Institute	RRID: CVCL_7672
SKMES1	ATCC	HTB-58; RRID: CVCL_0630
H1650	ATCC	CRL-5883; RRID: CVCL_1483
HCC4006	ATCC	CRL-2871; RRID: CVCL_1269
H2170	ATCC	CRL-5928; RRID: CVCL_1535
D54 MG	ATCC	RRID: CVCL_5735
D270 MG	ATCC	RRID: CVCL_S751
U87 MG	ATCC	HTB-14; RRID: CVCL_0022
U118 MG	ATCC	HTB-15; RRID: CVCL_0633
HT29	ATCC	HTB-38; RRID: CVCL_0320
SW620	ATCC	CCL-227; RRID: CVCL_0547
BT549	ATCC	HTB-122; RRID: CVCL_1092
MDA231	ATCC	HTB-26; RRID: CVCL_0062
MDA436	ATCC	HTB-130; RRID: CVCL_0623
PC-3	ATCC	CRL-1435; RRID: CVCL_0035
LNCaP clone FGC	ATCC	CRL-1740; RRID: CVCL_1379
A375	ATCC	CRL-1619; RRID: CVCL_0132
MAMEL65	ATCC	RRID: CVCL_A200
SKMEL28	ATCC	HTB-72; RRID: CVCL_0526
OE19	Sigma-Aldrich	Cat# 96071721; RRID: CVCL_1622
A2780	Sigma-Aldrich	Cat# 93112519; RRID: CVCL_0134
CAOV3	ATCC	HTB-75; RRID: CVCL_0201
IGROV1	Sigma-Aldrich	Cat# SCC203; RRID: CVCL_1304
OVCAR8	Creative Biolabs	Cat# IOC-ZP305; RRID: CVCL_1629

**Software and algorithms**		

Adobe Illustrator	Adobe	http://www.adobe.com/products/illustrator.html; RRID: SCR_010279
Biorender	Biorender	http://biorender.com; RRID: SCR_018361
GraphPad Prism	GraphPad Software	https://www.graphpad.com/; RRID: SCR_002798
MaxQuant	Max-Planck-Institute of Biochemistry	https://www.maxquant.org/; RRID: SCR_014485
MedCalc	MedCalc Software	https://www.medcalc.org/; RRID: SCR_015044
R Project for Statistical Computing	The R Foundation	https://www.R-project.org/; RRID: SCR_001905
SAS JMP Pro	JMP Statistical Discovery	https://www.jmp.com/en_us/home.html; RRID: SCR_022199
Vancouver Raman Algorithm	Zhao et al.^72^	N/A
WITec Project FIVE	Oxford Instruments	N/A

### Experimental model and study participant details

#### Human subjects

All work involving human samples was approved by the QIMR Berghofer Human Ethics committee under P2180. The retrospective discovery cohort of 764 samples consisted of 250 healthy subjects and 514 cancer patients (139 NSCLC, 57 glioblastoma, 42 colorectal, 30 prostate, 100 melanoma, 19 gastric, 98 esophageal and 29 SCLC patients). The median age of both healthy subjects and cancer patients at diagnosis was 65.5 (range 21–90). The distribution of gender was 113 (45%) females and 137 (55%) males in healthy subjects, and 137 (26.7%) females, 375 (72.9%) males and 2 (0.4%) unknown in cancer patients. The independent prospective confirmation cohort of 68 samples included 27 patients with benign lung diseases and 41 patients with early-stage lung cancer, sourced from the ongoing ACTRN12618001789257 trial. The median age of patients with benign lung diseases was 71 (range 46–84), and of patients with early-stage lung cancer was 70 (range 48–83). The distribution of gender was 15 (56%) females and 12 (44%) males in patients with benign lung diseases, and 20 (49%) females and 21 (51%) males in patients with early-stage lung cancer. Ethics approval for 12 pre- and post-surgery NSCLC patient samples was obtained from the Metro South Health District Human Research Ethics Committee under the National Health and Medical Research Council guidelines (HREC/11/QPAH/331) to collect samples from the Princess Alexandra Hospital. These 12 patients were treatment-naive and without prior cancer diagnosis within the last 5 years. The median age was 66.5 (range 52–76). The distribution of gender was 4 (33.3%) females and 8 (66.7%) males. Ethics approval of the additional 18 prostate cancer patients was obtained from The University of Queensland Institutional Human Research Ethics Committee (Approval No. 2004000047), and Royal Brisbane & Women’s Hospital Human Research Ethics Committee (Ref No. 1995/088B). Informed consent was obtained from all subjects prior to sample collection, and methods pertaining to clinical samples were carried out in accordance with approved guidelines. Gender, age and clinical information for all participants is provided in Tables S3–S5. Where applicable, gender and age were considered as covariates in the logistic regression models used to differentiate patient groups.

#### Cell lines

Cell line authentication was carried out using STR profiling for the panel of established cell lines used in this study. Isogenic immortalized normal human bronchial epithelial cells (HBECs, 30KT) transformed with p53 knockdown and KRAS v12 overexpression (30KT^p53/KRAS^) were a gift from Dr. Jill Larsen.^70^^,^^71^ HBECs were cultured in keratinocyte serum free medium (KSFM), supplemented with EGF (5 ng/mL) and bovine pituitary extract (50 mg/L), at 37°C in 5% CO_2_. All other cell lines (HOSE 6.3 and HOSE 17.1; Lung [SKMES1, H1650, HCC4006, H2170]; Brain [D54, D270, U87, U118]; Colorectal [HT29, SW620]; Breast [BT549, MDA231, MDA436]; Prostate [LNCaP]; Melanoma [A375, MAMEL65, SKMEL28]; Esophageal [OE19]; Ovarian [A2780, CAOV3, IGROV1, OVCAR8]) were maintained in DMEM or RPMI supplemented with 5% fetal bovine serum, 100 U/mL penicillin and 100 mg/mL streptomycin and incubated at 37°C in 5% CO_2_. Cell conditioned media (CCM) was collected from cells cultured in serum-free media. CCM was collected from HBECs in KSFM depleted of bovine sEVs through overnight centrifugation at 100,000 *g*_avg_. All cell lines were routinely tested for mycoplasma contamination.

### Method details

#### sEV isolation

sEVs were isolated and analyzed as previously described.^73^^,^^74^ Blood samples were collected in both serum and EDTA plasma tubes. For serum preparation, samples were centrifuged at 3,000 rpm for 10 min, the upper serum layer was removed, and 500 μL aliquots were stored at −80°C. Platelet-depleted EDTA plasma was prepared using a double-spin procedure, and the resulting plasma was stored at −80°C until use. For the isolation of sEVs from human plasma, plasma was thawed at room temperature and prepared by removing remaining platelets and large vesicles by centrifugation at 1,500 *g* and 10,000 *g*, for 10 and 20 min respectively. Prepared plasma was overlaid on Izon qEV Original 70 nm (Izon Science) size exclusion chromatography (SEC) columns followed by elution with 10 mM PBS. Following the void volume, the following sEV-enriched fractions were concentrated to ≤50 μL using Amicon Ultra-4 10 kDa MWCO columns (Merck) at 3,500 × g for approximately 45 min at 4°C. The concentrated sEV isolates were aliquoted and stored at −80°C. All clinical samples were processed and analyzed in a blinded fashion for the SERS analysis: sample identifiers were anonymized prior to analysis, assays were performed without knowledge of clinical status by the SERS operator, and decoding occurred only after data acquisition and statistical evaluation.

For sEV mass spectrometry analysis, CCM was centrifuged at 300 *g* for 10 min at 4°C and filtered through 0.22 μm filters to remove floating cells and large extracellular vesicles. Clarified CCM was then concentrated to 500 μL and overlaid on a discontinuous iodixanol density gradient and centrifuged for 16 h at 100,000 *g*_avg_ at 4°C. sEV containing fractions were diluted to 20 mL in 10 mM PBS and centrifuged at 100,000 *g*_avg_ at 4°C for 2 h. The resulting pellet was resuspended in 10 mM PBS and stored at −80°C until use. All other sEV isolations from *in vitro* CCM were clarified and concentrated as described above and then purified using Izon qEV Original 70 nm (Izon Science) SEC columns.

#### Tunable resistive pulse sensing (TRPS)

The concentration and size distribution of particles was analyzed with TRPS (qNano, Izon Science Ltd) using an NP100 nanopore at a 45 mm stretch. The concentration of particles was standardized using multi-pressure calibration with 70 nm carboxylated polystyrene beads at a concentration of 1.5×10^11^ particles/mL.

#### Western blot

Western blots were performed as previously described.^74^ Briefly, sEV isolations were lysed in reducing sample buffer [0.25 M Tris–HCl (pH 6.8), 40% glycerol, 8% SDS, 5% 2-mercaptoethanol and 0.04% bromophenol blue] or non-reducing sample buffer (without 2-mercaptoethanol) and boiled for 10 min at 95°C. Proteins were resolved by SDS-PAGE (SDS-polyacrylamide gel electrophoresis), transferred to polyvinylidene fluoride membranes, blocked in 5% non-fat powdered milk in PBS-T (0.5% Tween 20) and probed with antibodies. All proteins were resolved under fully denaturing and reducing conditions, apart from CD63, which was resolved under non-reducing conditions. Protein bands were detected using X-ray film and enhanced chemiluminescence reagent (Amersham ECL Select).

#### ELISA

Quantification of THBS1, NID1, PTX3 and VCAN in sEVs was performed using commercial ELISA kits (THBS1, R&D Systems, Cat# DY3074; NID1, R&D Systems, Cat# DY2570; PTX3, R&D Systems, Cat# DY1826; VCAN, Novus Biologicals, Cat# NBP2-75353) following the manufacturer’s instructions. Absorbance was measured at 450 nm using a microplate reader, and concentrations were calculated based on standard curves.

#### Nano flow cytometry

The concentrations and size distributions of purified sEVs were analyzed using nanoflow cytometry (NanoFCM Inc.) following the manufacturer’s instructions. For robust analysis, samples were diluted in filtered PBS to make sure that 4000–8000 events were recorded in one minute. 250 nm fluorescent silica microspheres (NanoFCM Inc.) and silica nanosphere cocktails 68–155 nm (NanoFCM Inc.) were used as references for the calculation of particle concentrations and size distributions, respectively.

#### MicroBCA

Total protein concentration of sEV samples was determined using the Micro BCA Protein Assay Kit (Thermo Scientific) according to the manufacturer’s instructions. Absorbance was measured at 562 nm, and protein concentrations were calculated using a BSA standard curve.

#### Mass spectrometry

sEV preparations were reduced by the addition of 10 mM dithiothreitol (4°C 1 h, 22°C 2 h) in the presence of 2% SDS, protease inhibitors (SigmaAldrich, Cat# P8340) and 50 mM Tris-HCl (pH 8.8). Samples were then alkylated by the addition of iodoacetamide to 25 mM (22°C 1 h) and methanol co-precipitated overnight at −20°C with trypsin (1:100 enzyme:substrate). Pellets were resuspended in 10% acetonitrile, 40 mM ammonium bicarbonate and digested at 37°C for 8 h with further trypsin added after 2 h (1:100 enzyme:substrate).

LC-MS analysis of acidified digests (trifluoroacetic acid) was performed by interfacing a NanoAcquity UPLC (Waters) in front of an Elite Orbitrap ETD mass spectrometer (Thermo Fisher Scientific). 2 µg of digest was loaded onto a 20 mm × 180 μm Symmetry C18 trap (Waters) and separated over 120 min on a 200 mm × 75 μm, BEH130 1.7 μm column (Waters) using a series of linear gradients (buffer A: aqueous 0.1% formic acid; buffer B: 0.1% formic acid in acetonitrile) 2% B to 5% B over 5 min, 30% B over 75 min, 50% B over 10 min 95% B over 5 min and hold for 6 min, re-equilibrate in 2% B. Eluate from the column was introduced into the mass spectrometer through a 10 μm P200P coated silica emitter (New Objective) and Nanospray-Flex source (Proxeon Biosystems A/S). Source voltage 1.8 kV, heated capillary temperature 275°C, using a top 15 method MS acquired in the orbitrap at 120,000 resolution AGC 1E6, MS2 in the ion-trap AGC 1E4, 50 ms maximum injection time. MS1 lock mass of 445.120024 was used.

Protein identification and label-free quantification were performed using MaxQuant (version 1.5.7.4^75^) as previously described.^12^ Briefly, MaxQuant was used to assign peptide sequences to tandem mass spectra, assign peptides to proteins according to the principle of parsimony and perform label-free quantification. The complete proteome for *Homo sapiens* (70,956 canonical sequences downloaded from www.uniprot.org on 12 January 2017) was used for the MaxQuant (RRID:SCR_014485) database search. A false discovery rate (FDR) threshold of 0.01 was used for peptide and protein identifications.

#### SERS nanotag barcode synthesis

SERS nanotag barcodes were prepared by functionalizing 60 nm gold nanoparticles with Raman reporters and target-specific antibodies, according to the previous method.^76^ To synthesize 60 nm gold nanoparticles, gold (III) chloride trihydrate (Sigma-Aldrich) was reduced by sodium citrate in the boiling condition.^77^ Following this, 1 mL of citrate-reduced 60-nm gold nanoparticles were then incubated with 10 μL of 1 mM Raman reporters and 2 μL of 1 mM dithiobis(succinimidyl propionate) (Thermo Fisher Scientific) for 5 h, 1 μg of primary antibodies for 0.5 h, and 0.1% (w/v) bovine serum albumin for 0.5 h at room temperature, sequentially. Centrifugation-based washing steps were performed between each incubation process to remove the free reagents. The synthesized SERS nanotag barcodes were then stored at 4°C until use.

SERS nanotag barcodes were synthesized with the Raman reporter-antibody pair of 4-mercaptopyridine (MPY)-THBS1; 4-mercaptobenzoic acid (MBA)-NID1; 2,3,5,6-tetrafluoro-MBA (TFMBA)-PTX3; 5,5′-dithiobis (2-nitrobenzoic acid) (DTNB)-VCAN, respectively. The signal spectra of these SERS nanotag barcodes show characteristic peaks at 1000 cm^−1^ (MPY), 1075 cm^−1^ (MBA), 1375 cm^−1^ (TFMBA), 1335 cm^−1^ (DTNB), respectively (Figure S7C). For the characterization of captured sEVs on the electrode, SERS nanotags including MBA-CD9 and TFMBA-CD63 were synthesized.

#### Microfluidic device fabrication

The microfluidic device (Figure S6) was fabricated using standard photolithography and as previously reported.^78^ In brief, a 5-inch chrome mask was prepared that carried the device design and consisted of an array of 4 rows with 7 asymmetric pairs of electrodes per row (total of 28 electrodes per device). Each pair of electrodes was made of an inner circular electrode (diameter = 1,000 μm) that was separated by 1,000 μm from the outer ring-shaped electrode (width = 120 μm). The chrome mask was then inserted into an EVG 620 mask aligner (EV Group, Austria). A clean 4-inch Boroflat 33 glass wafer (1 mm thick, Bonda Technology PTE LTD, Singapore) was coated with negative photoresist AZnLOF 2020 (Microchemicals GmbH, Germany) at 3,000 rpm for 30 s, baked at 110°C for 2 min, and placed in the mask aligner for UV exposure at a constant dose of 200 mJ/cm^2^. After exposure, the wafer was baked at 110°C for 1 min, cooled to room temperature and developed for 45 s in AZ 726MIF. After rinsing with purified water and drying with a stream of nitrogen, the developed wafer was subjected to plasma cleaning (Oxford Instruments, United Kingdom) for 30 s to remove photoresist residues. Subsequently, the wafer was transferred to a physical vapor deposition system Temescal (Ferrotec, U.S.A) for coating of 10 nm Ti and 200 nm Au. For lift off, the gold-coated wafer was immersed in Remover PG (Microchemicals GmbH, Germany) overnight and rinsed with isopropanol and water to reveal the electrode array.

To hold the sample on each pair of asymmetric electrodes, a well structure made of polydimethylsiloxane (PDMS) was prepared. PDMS elastomer and curing agent (SYLGARD 184, Dow, U.S.A) were mixed at a ratio of 1:10 and cured at 80°C for 20 min. Holes of 6 mm diameter were then punched to the cured PDMS and the PDMS was aligned on the glass wafer with electrode array. Thermal bonding of PDMS on glass wafer overnight at 65°C overnight completed the device fabrication process.

#### Device functionalization and operation

We conjugated the anti-THBS1 antibody to the gold microelectrode surface by using the chemical cross-linker dithiobis(succinimidyl propionate) (DSP). Briefly, 30 μL of 4 mg/mL DSP in dimethyl sulfoxide were pipetted to the clean gold microelectrode surfaces and incubated for 30 min. After three washes with ethanol and 10 mM PBS, 10 μg/mL anti-THBS1 antibody in 10 mM PBS was incubated for 2 h. Subsequently, the microelectrode wells were blocked by adding a solution of 3% bovine serum albumin for 1 h. All steps were performed at room temperature.

50 μL of sample containing 0.6 μg of total sEV protein (i.e., diluted purified sEVs from plasma in 10 mM PBS) was added to the microelectrode wells and incubated for 45 min under previously optimized alternating current electric field conditions of 800 mV and 500 Hz.^78^ Subsequently, 30 μL of SERS nanotags were added and incubated for 20 min using the same conditions as above. The wells were washed three times with 10 mM PBS in-between incubation steps.

#### On-chip profiling of sEVs

SERS mapping was performed on an area of 60 μm × 60 μm of microelectrodes, using the WITec Alpha300 R microspectrometer configured with the highly sensitive EMCCD. The SERS signals of samples were excited under the 633 nm laser with 4 mW laser power for 0.05 s integration time and collected with a 20× microscope objective.

#### Transmission electron microscopy (TEM)

For TEM analysis of sEVs, 2.5 μL of sEVs (1 × 10^11^ particles/mL) were fixed with an equal volume of 2% glutaraldehyde for 30 min at room temperature. 5 μL of fixed sample was loaded on Formvar/carbon-coated electron microscopic grids (Electron Microscopy Science) and incubated for 15 min. Excess liquid was removed by blotting. The grid was washed three times by brief contact with 100 μL of Milli-Q water, followed by blotting to remove excess liquid. To contrast the sample, the grid was placed on 30 μL of 2% uranyl acetate (w/v) for 5 min and excess fluid was removed by blotting gently. Grids were left to air dry and observed using transmission electron microscopy (Hitachi HT7700) at 100 kV.

For TEM analysis of gold nanoparticles, particles were diluted in water and loaded on Formvar/carbon-coated electron microscopic grids (Electron Microscopy Science). Grids were left to air dry and observed using transmission electron microscopy (Hitachi HT7700) at 100 kV.

#### Scanning electron microscope (SEM)

Gold nanoparticles were diluted in water and loaded on a silicon slice mounted on an SEM stub using carbon tabs. Samples were baked in a vacuum oven at 70°C overnight and plasma cleaned before SEM analysis. SEM imaging was conducted using a JEOL JSM-7800F FE-SEM microscope with 10 kV voltage and a working distance of 10 mm.

### Quantification and statistical analysis

GraphPad Prism version 6.0 (RRID:SCR_002798), R version 3.3.1, MedCalc (RRID:SCR_015044) version 16.8.4 and JMP Pro version 15.1.0, were used for all calculations. Unpaired Student’s *t* test was used to calculate the difference in expression values of proteins from sEVs *in vitro*. Subcellular localization of proteins was generated through IPA (QIAGEN Inc). A negative-binomial exact test was used to assess the mass spectrometry data, where the Benjamini-Hochberg adjustment was applied to control the FDR. A nominal logistic regression model was used to classify healthy and cancer cases. Differences with *p*-values less than 0.05 were considered significant (∗*p* < 0.05, ∗∗*p* < 0.01, ∗∗∗*p* < 0.001), with the exception of an FDR threshold of 0.001.

### Additional resources

The independent prospective confirmation is sourced from the ongoing ACTRN12618001789257 trial registered at Australian New Zealand Clinical Trials Registry (ANZCTR) (https://anzctr.org.au/Trial/Registration/TrialReview.aspx?ACTRN=12618001789257).

## Supplemental information

Document S1. Figures S1–S12 and Tables S1 and S3–S5

Table S2. Differentially expressed proteins between normal and transformed HBEC sEVs, related to Figure 2

Document S2. Article plus supplemental information
