# Supplementary material for: Early-stage multi-cancer detection through a plasma extracellular vesicle protein signature
Source: Cell Rep Med. 2026 Mar 24;7(4):102694. doi: 10.1016/j.xcrm.2026.102694 (PMC13130687; doi:10.1016/j.xcrm.2026.102694)
Supplement: Document S1. Figures S1–S12 and Tables S1 and S3–S5 [file mmc1.pdf]

Cell Reports Medicine, Volume *7*

Supplemental information

Early-stage multi-cancer detection through a plasma extracellular vesicle protein signature

Richard J. Lobb, Quan Zhou, David Fielding, Kekoolani S. Visan, Alain Wuethrich, Jing Wang, Youran Hu, Emma L. Norris, Marcus L. Hastie, Sarah Everitt, Brielle Parris, Lauren G. Aoude, Vanessa F. Bonazzi, Elizabeth Nixon, Jennifer Mooi, Kevin M. Koo, Kenneth O'Byrne, Arutha Kulasinghe, Rayleen V. Bowman, Ian A. Yang, Niall M. Corcoran, Christopher M. Hovens, Michael MacManus, Jeffrey J. Gorman, Bryan W. Day, Gunter Hartel, David C. Whiteman, Niall Tebbutt, John M. Mariadason, Kwun M. Fong, Andrew P. Barbour, Matt Trau, and Andreas Möller

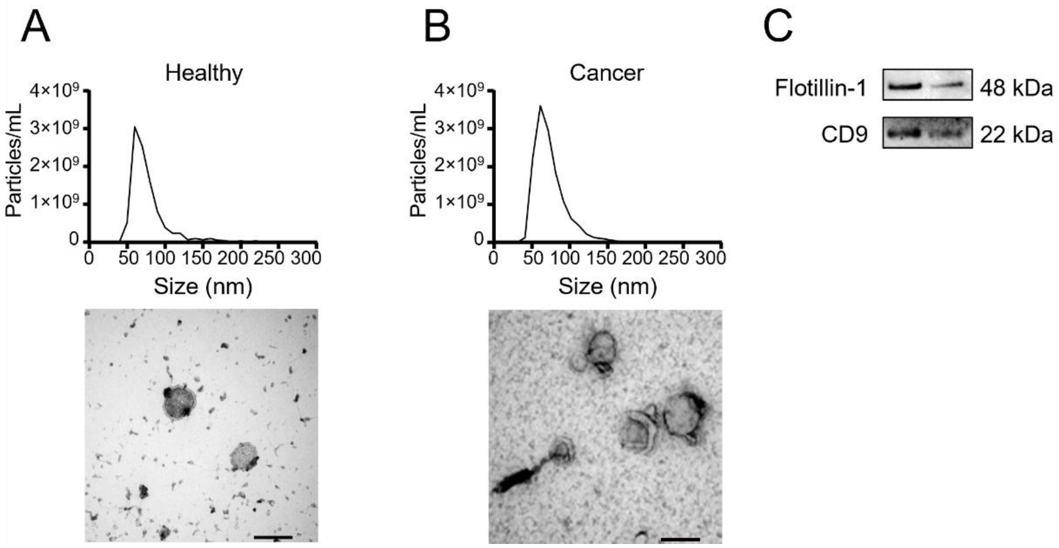

Figure S1. Isolation of sEVs from patient samples. Related to Figure 3.

Representative size distribution analysis and TEM images (size bar 200 nm) of sEVs isolated from the healthy control (A) and cancer patient (B).

(C) Western blot of isolated sEVs from representative patients.

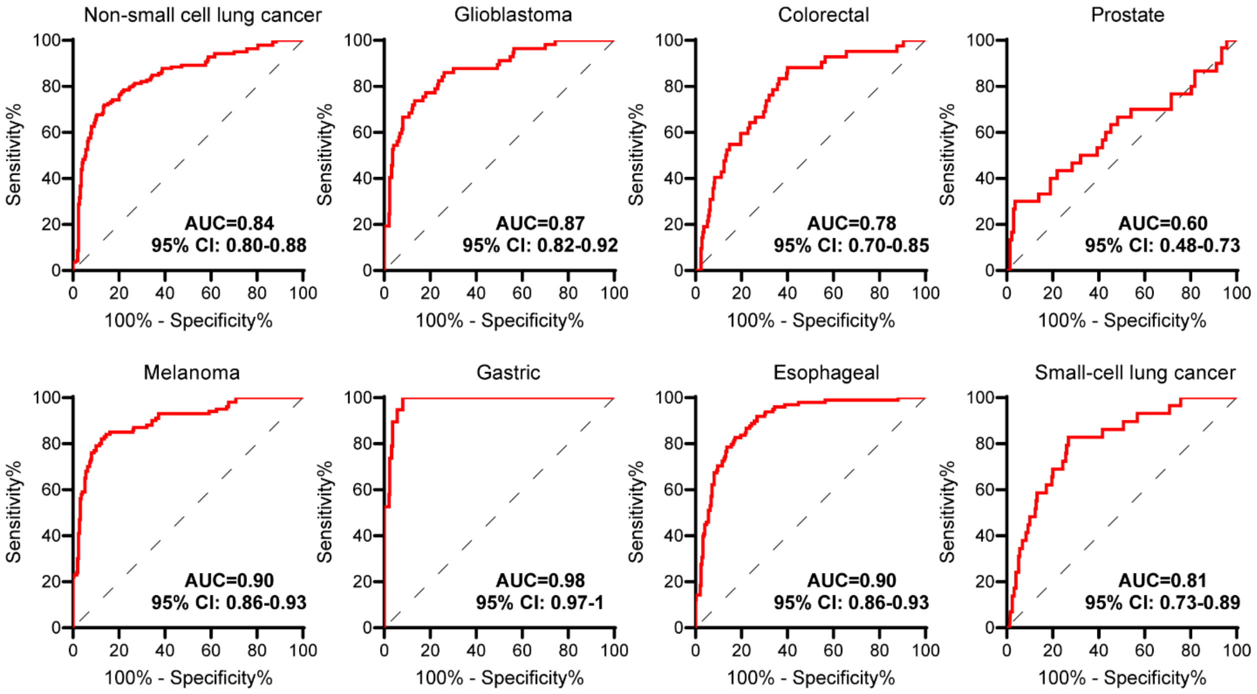

Figure S2. ROC curves for THBS1 in different cancer types. Related to Figure 3.

ROC curve analysis of classification of non-small cell lung cancer (n=139), glioblastoma multiforme (n=57), colorectal cancer (n=42), prostate cancer (n=30), melanoma (n=100), gastric cancer (n=19), esophageal cancer (n=98) and small-cell lung cancer (n=29) samples against healthy controls for THBS1 showed AUC of 0.60 to 0.98.

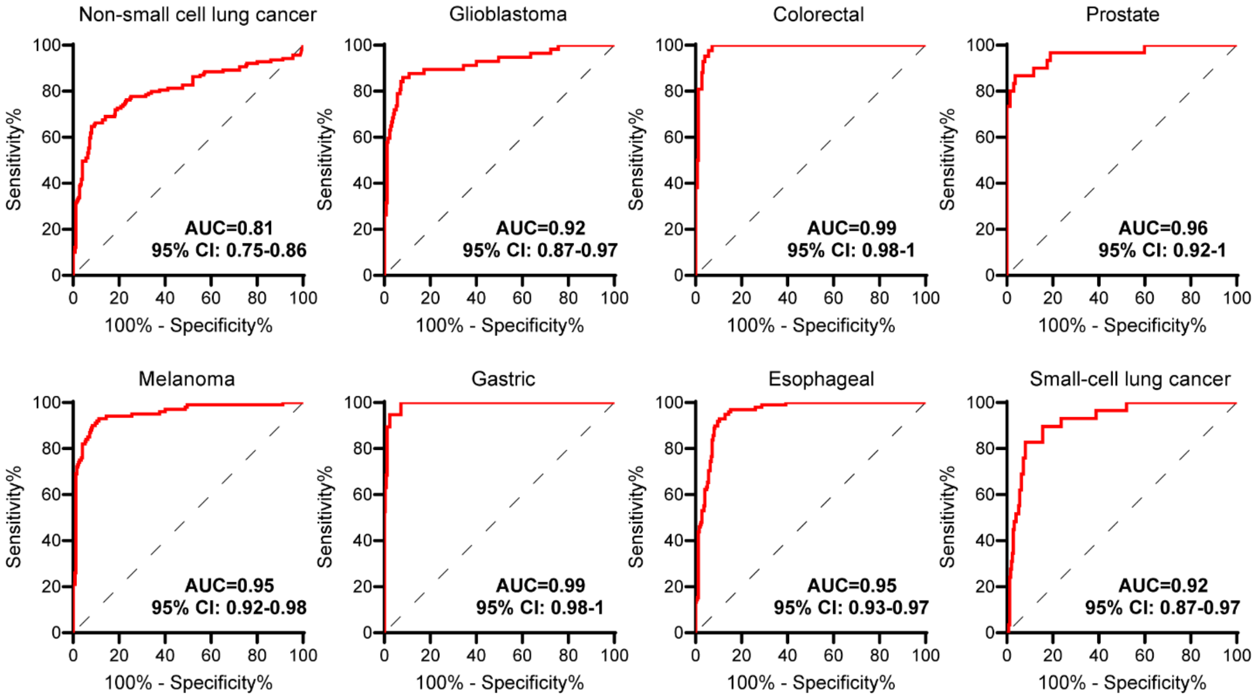

Figure S3. ROC curves for NID1 in different cancer types. Related to Figure 3.

ROC curve analysis of classification of non-small cell lung cancer (n=139), glioblastoma multiforme (n=57), colorectal cancer (n=42), prostate cancer (n=30), melanoma (n=100), gastric cancer (n=19), esophageal cancer (n=98) and small-cell lung cancer (n=29) samples against healthy controls for NID1 showed AUC of 0.81 to 0.99.

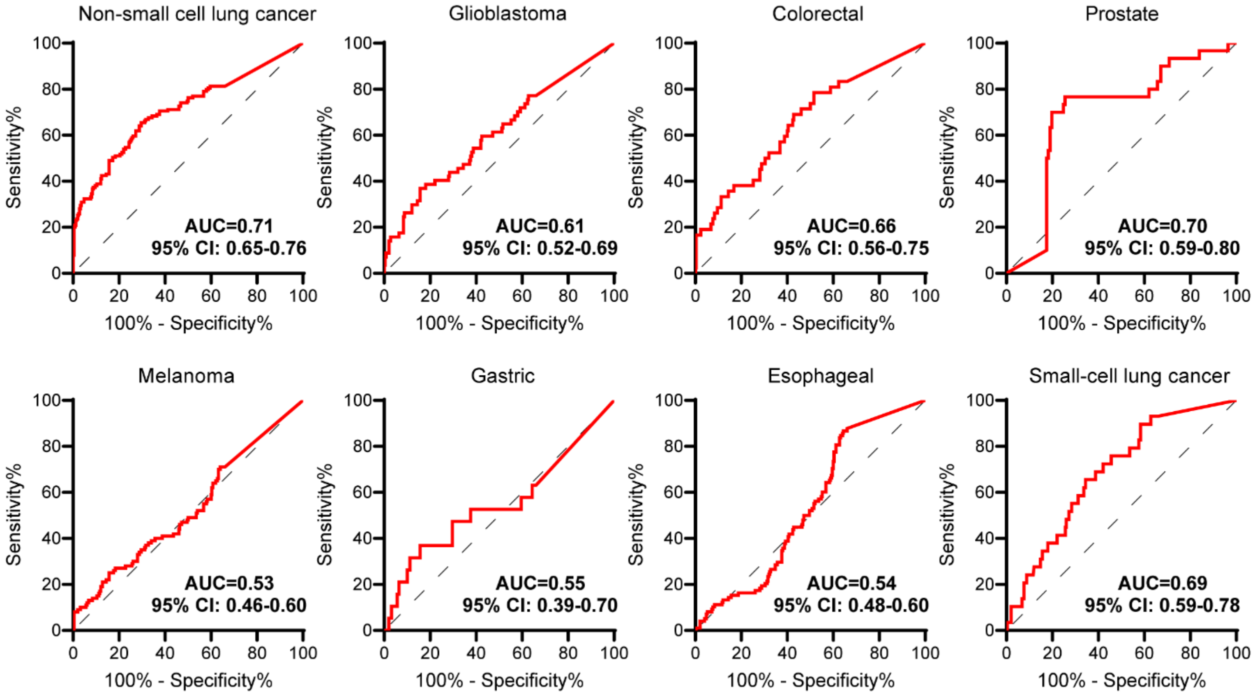

Figure S4. ROC curves for PTX3 in different cancer types. Related to Figure 3.

ROC curve analysis of classification of non-small cell lung cancer (n=139), glioblastoma multiforme (n=57), colorectal cancer (n=42), prostate cancer (n=30), melanoma (n=100), gastric cancer (n=19), esophageal cancer (n=98) and small-cell lung cancer (n=29) samples against healthy controls for PTX3 showed AUC of 0.53 to 0.71.

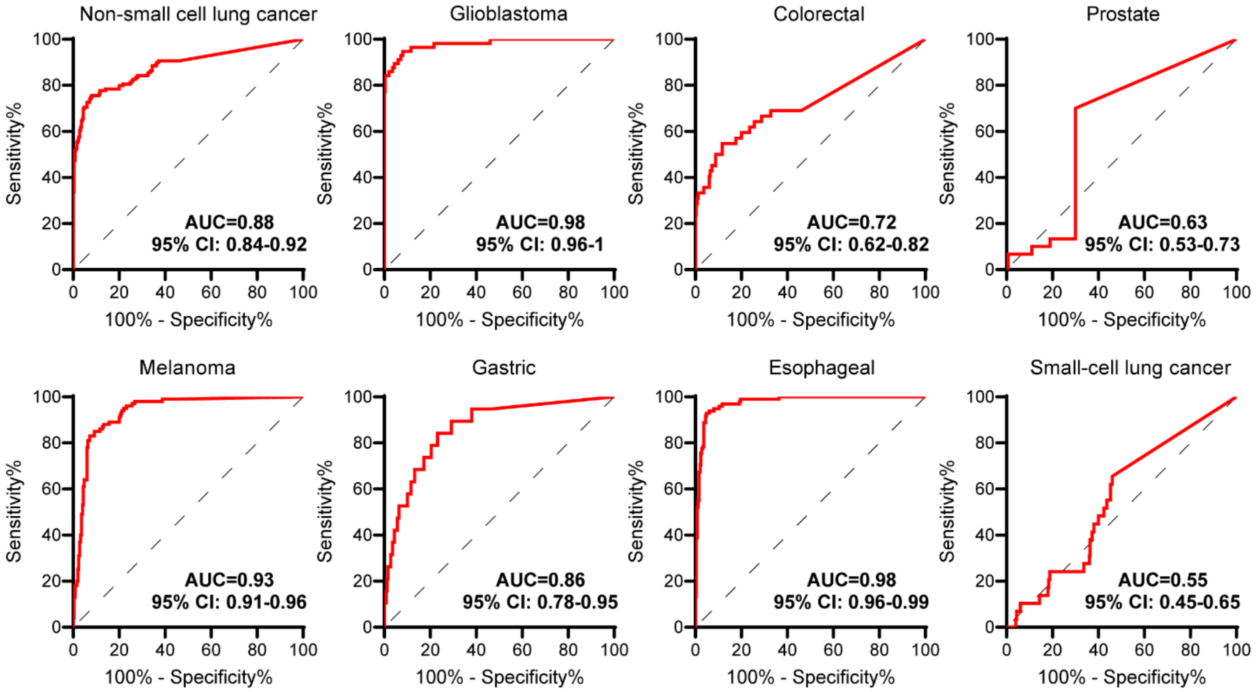

Figure S5. ROC curves for VCAN in different cancer types. Related to Figure 3.

ROC curve analysis of classification of non-small cell lung cancer (n=139), glioblastoma multiforme (n=57), colorectal cancer (n=42), prostate cancer (n=30), melanoma (n=100), gastric cancer (n=19), esophageal cancer (n=98) and small-cell lung cancer (n=29) samples against healthy controls for VCAN showed AUC of 0.55 to 0.98.

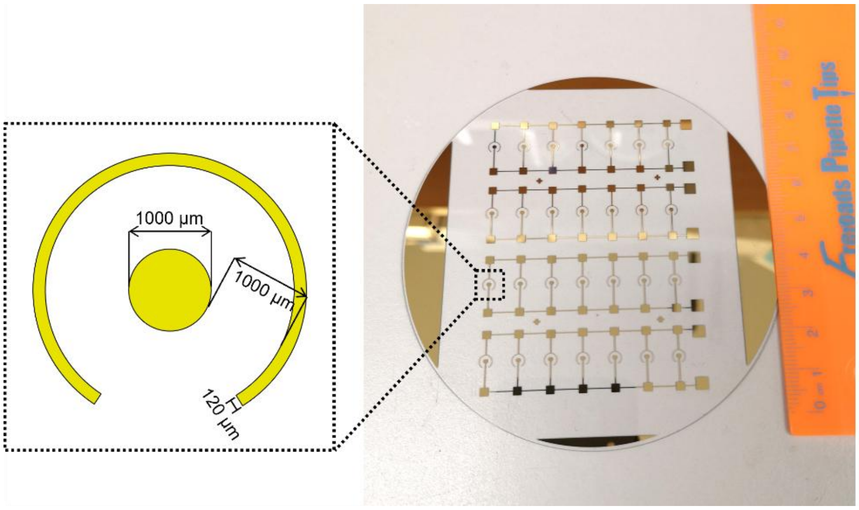

Figure S6. Geometry of the multiplex microfluidic device. Related to STAR Methods.

The device is composed of 28 asymmetrical gold microelectrodes, allowing parallel analysis of 28 samples.

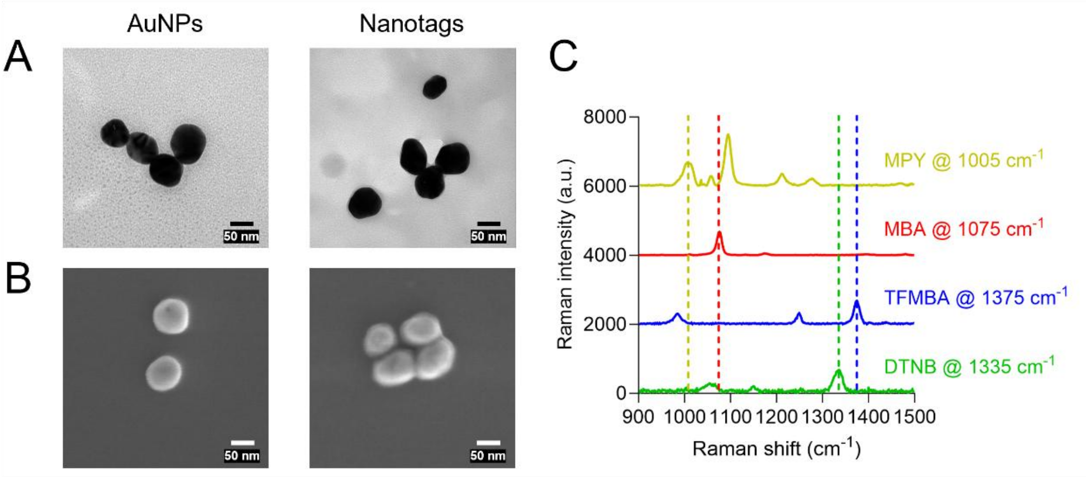

Figure S7. Characterization of gold nanoparticles and functionalized nanotags. Related to STAR Methods.

TEM (A) and SEM (B) images of gold nanoparticles (AuNPs) and functionalized SERS nanotags. Size bar 50 nm.

(C) Raman spectra of SERS nanotags (MPY, 1005 cm^{-1}, yellow; MBA, 1075 cm^{-1}, red; TFMBA, 1375 cm^{-1}, blue; DTNB, 1335 cm^{-1}, green). a.u., arbitrary units.

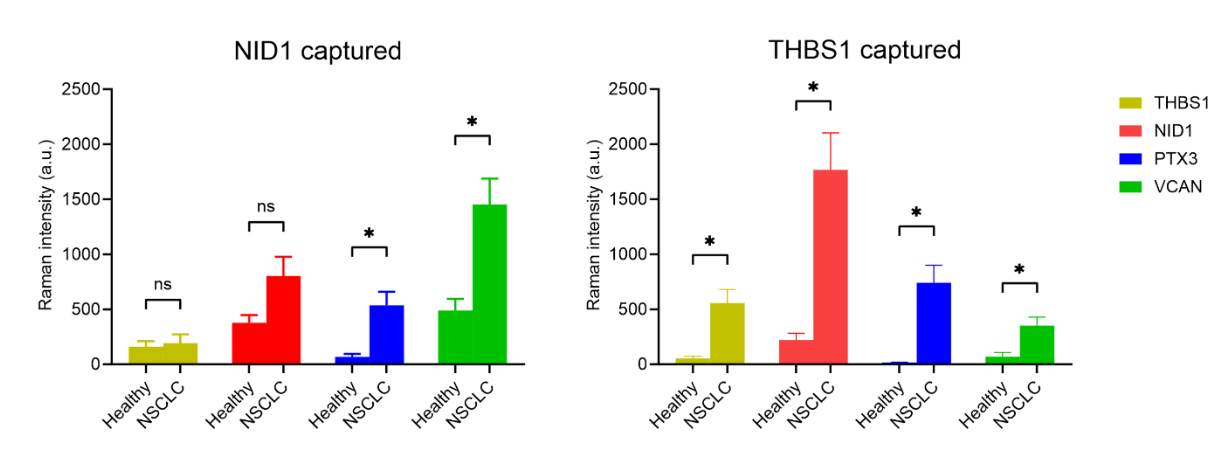

Figure S8. Comparison of capture antibodies against NID1 or THBS1. Related to Figures 5 and 6.

sEVs derived from healthy individuals (n=3) and NSCLC patients (n=3) were captured by anti-NID1 or anti-THBS1 antibody on the electrode and labeled with 4-protein sEV biomarker panel. Data are represented as mean ± standard error of three independent experiments. a.u., arbitrary units. Two-tailed Welch's t-tests were performed. * $p < 0.05$; ns, not significant.

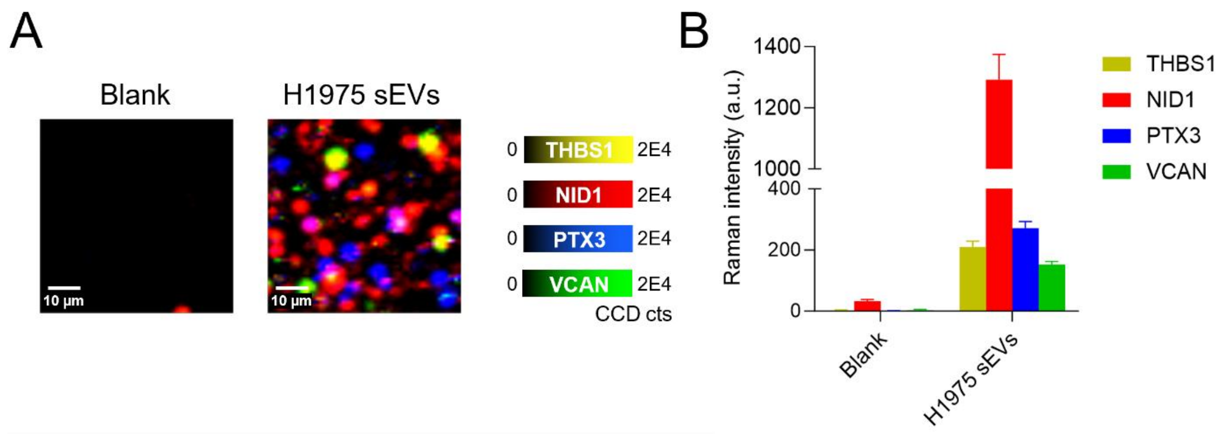

Figure S9. Specificity of the multiplex SERS assay. Related to Figures 5 and 6.

(A) Representative false-color SERS spectral images of blank control (1% (w/v) bovine serum albumin (BSA) in PBS) and H1975-derived sEVs. Size bar 10 μm.

(B) Higher signals in H1975-derived sEVs and negligible signals in blank control indicated that sEVs were specifically analyzed. Data are represented as mean ± standard error of three independent experiments. a.u., arbitrary units.

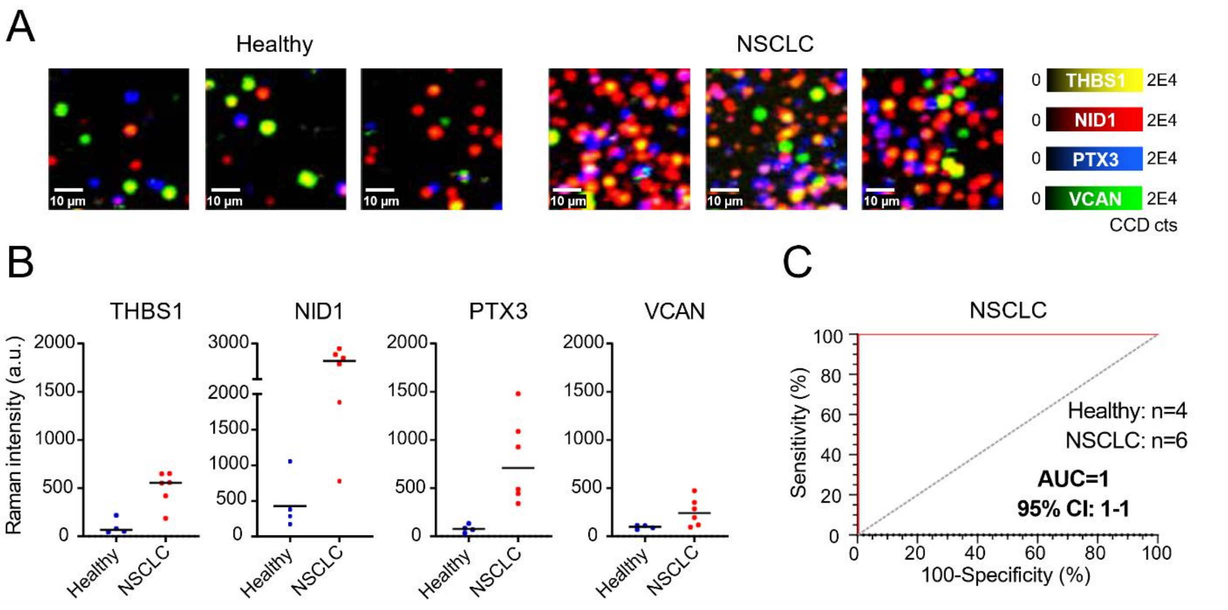

Figure S10. Application of the multiplex microfluidic device in healthy individuals and late-stage NSCLC patients. Related to Figures 5.

(A) Representative false-color SERS spectral images demonstrating an enrichment of THBS1, NID1, PTX3, VCAN in late-stage NSCLC patients compared to healthy individuals. Size bar 10 μm.

(B) The Raman intensity of each biomarker THBS1, NID1, PTX3, VCAN in healthy individuals (n=4) and late-stage NSCLC patients (n=6). a.u., arbitrary units. Samples were measured in triplicate. Lines in dot plots represent median values.

(C) ROC curve of logistic regression classification with an AUC of 1 indicating a perfect diagnostic capability.

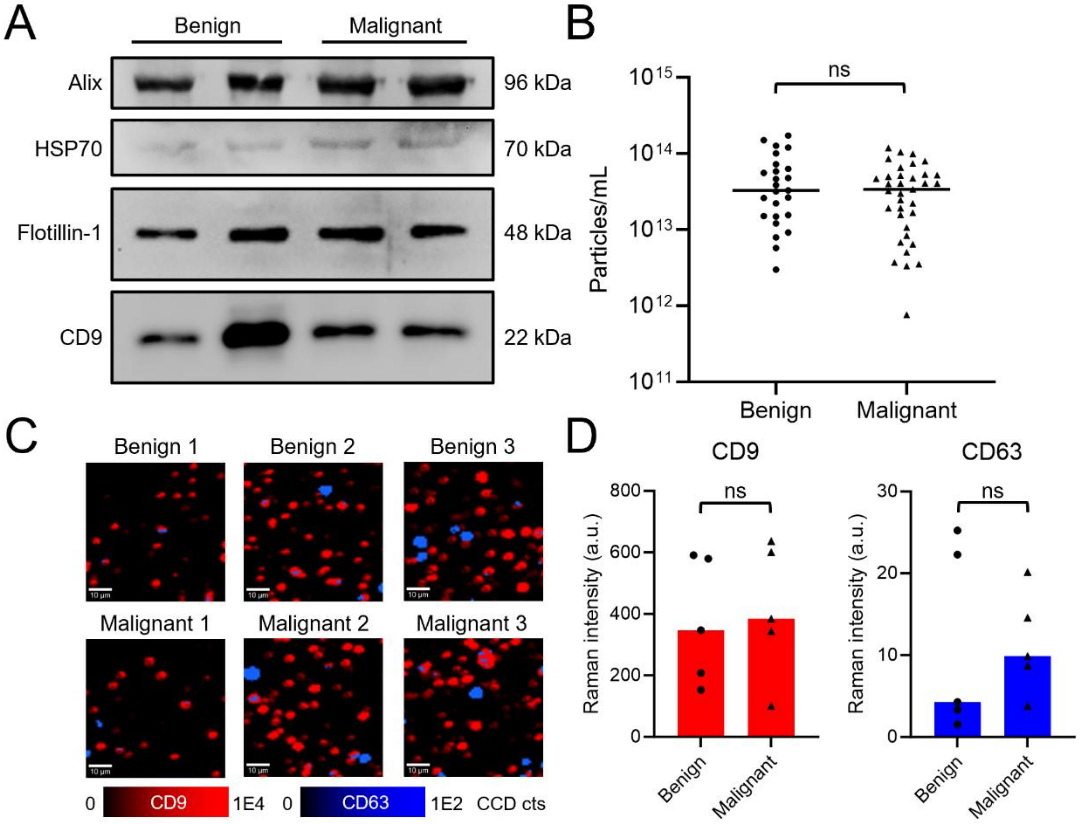

Figure S11. Characterization of sEVs from patients with benign lung diseases and early-stage NSCLC patients. Related to Figure 5.

(A) Western blot of sEVs from representative benign and malignant patients demonstrated the presence of sEV proteins Alix, HSP70, Flotillin-1 and CD9.

(B) No significant differences in sEV concentrations between benign (n=25) and malignant (n=35) patients after SEC isolation from 500 µL of plasma. Lines in dot plots represent median values.

(C) Representative false-color SERS spectral images demonstrated the expression of CD9 and CD63, indicating that sEVs from benign and malignant patients were captured by anti-THBS1 antibody on the electrode. Size bar 10 µm.

(D) No significant differences in the Raman intensities of CD9 and CD63 in sEVs from representative benign (n=5) and malignant (n=5) patients. Samples were measured in triplicate. Bars in dot plots represent median values. a.u., arbitrary units. Two-tailed Welch's t-tests were performed. ns, not significant.

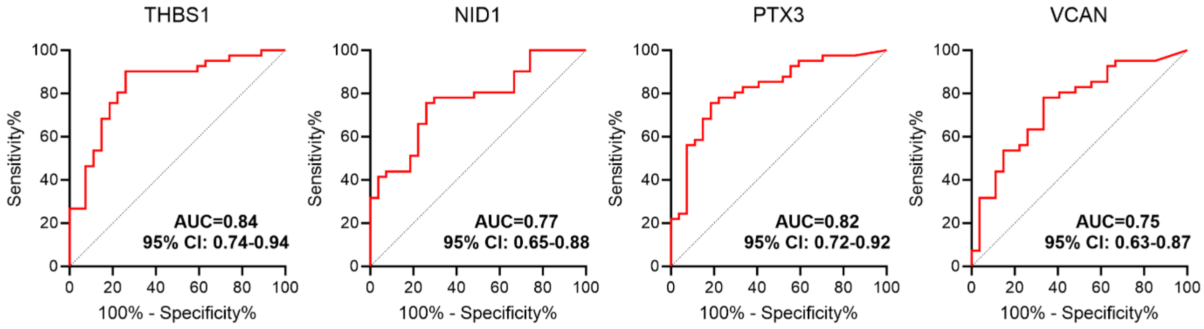

Figure S12. Individual ROC curves for four markers in patients with benign lung diseases (n=27) and early-stage NSCLC patients (n=41). Related to Figure 5.

AUC values range from 0.75 to 0.84.

Table S1. Label-free mass spectrometry indicating the presence of sEV markers. Related to Figure 2.

Protein names	Gene names	Exclusive PSMs	30KT	30KT	30KT	$30KT^{(p53/KRAS)}$	$30KT^{(p53/KRAS)}$	$30KT^{(p53/KRAS)}$
Alix	PDCD6IP	746	28.59	30.12	29.08	29.67	29.56	29.89
CD9	CD9	129	28.61	30.22	29.26	29.56	28.81	29.37
CD63	CD63	39	24.21	21.25	21.21	26.64	26.33	26.46
CD81	CD81	206	26.19	28.24	26.92	27.92	27.7	28.29
Tumor susceptibility gene 101 protein	TSG101	127	25.17	26.87	26.14	26.96	26.96	27.29

Table S3. Clinical information of healthy controls and patients. Related to Figure 3.

Characteristics	Healthy controls	Non-small cell lung cancer	Glioblastoma	Colorectal cancer	Prostate cancer	Melanoma	Gastric cancer	Esophageal cancer	Small-cell lung cancer
**Total samples**	250	139	57	42	30	100	19	98	29
**Age**									
Median	65	68	60	68	Unknown	60	60	66	65
Range	21-90	31-90	21-81	41-86	Unknown	27-85	34-79	40-88	52-83
**Gender**									
F	113 (45%)	39 (28%)	21 (37%)	14 (33%)	NA	29 (29%)	7 (37%)	13 (13%)	14 (48%)
M	137 (55%)	100 (72%)	36 (63%)	28 (67%)	30 (100%)	71 (71%)	10 (53%)	85 (87%)	15 (52%)
Unknown	-	-	-	-	-	-	2 (10%)	-	-
**Stage**									
I	NA	52	NA	-	-	-	10	18	NA
II	NA	33	NA	-	-	-	4	34	NA
III	NA	33	NA	-	-	90	1	29	NA
IV	NA	1	NA	42	-	10	3	14	NA
Unknown	-	20	-	-	30	-	1	3	-

Table S4. Clinical information of benign and early-stage NSCLC patients. Related to Figure 5.

Characteristics	Benign	Stage I/II NSCLC
**Total samples**	27	41
**Age**		
Median	71	70
Range	46-84	48-83
**Gender**		
F	15 (56%)	20 (49%)
M	12 (44%)	21 (51%)

Table S5. Clinical information of pre- and post-surgery lung cancer patients. Related to Figure 6.

Patient ID	Gender	Age	Stage	Tumor
P1	M	52	NA	Squamous cell carcinoma, PD-L1: 30%, TMB: High (20 Mut/Mbp)
P2	M	52	IIIA	IIIA NSCLC - pleomorphic sarcomatoid carcinoma
P3	M	54	NA	Adenocarcinoma of lung T2a N0; EGFR neg (1ng of tissue) ALK/ROS-1 neg; PD-L1: <1%
P4	M	59	IB	IB squamous cell, PD-L1: 80%
P5	M	62	NA	EGFR
P6	F	66	IIB	IIB squamous cell, PD-L1: 0%
P7	F	68		–
P8	M	68	IB	Ib adenocarcinoma, EGFR/ALK/ROS-1/RAS/ERBB2 negative; BRAF positive PD-L1: 5%
P9	M	67	IA	IA2 adenocarcinoma, EGFR/ALK/ROS-1 neg, PD-L1: 0%
P10	M	68	IIB	IIB adenocarcinoma, EGFR/ALK/Ros-1 neg, PD-L1: 10%
P11	F	74	IA2	IA2 adenocarcinoma, EGFR/ALK/ROS-1/KRAS/ERBB2/MET neg, BRAF Gly469Val positive, PD-L1: 0%
P12	F	76	IB	Ib adenocarcinoma, EGFR/ALK/ROS-1/RAS/BRAF/ERBB2 negative; PD-L1: 0%
